# Decoding breast cancer tissue–stroma interactions using species-specific sequencing

**DOI:** 10.1186/s13058-015-0616-x

**Published:** 2015-08-13

**Authors:** Indira V. Chivukula, Daniel Ramsköld, Helena Storvall, Charlotte Anderberg, Shaobo Jin, Veronika Mamaeva, Cecilia Sahlgren, Kristian Pietras, Rickard Sandberg, Urban Lendahl

**Affiliations:** Department of Cell and Molecular Biology, Karolinska Institutet, von Eulers väg 3, SE-17177 Stockholm, Sweden; Ludwig Institute for Cancer Research, Karolinska Institutet, Stockholm, Sweden; Department of Medical Biochemistry and Biophysics, Karolinska Institutet, Stockholm, Sweden; Turku Centre for Biotechnology, University of Turku and Åbo Akademi University, Turku, Finland; Present address: Rheumatology Unit, Department of Medicine Solna, Karolinska Institutet, Stockholm, Sweden; Present address: Institute of Environmental Medicine, Karolinska Institutet, Stockholm, Sweden; Present address: Department of Laboratory Medicine, Medicon Village, Lund University, Lund, Sweden

## Abstract

**Introduction:**

Decoding transcriptional effects of experimental tissue–tissue or cell–cell interactions is important; for example, to better understand tumor–stroma interactions after transplantation of human cells into mouse (xenografting). Transcriptome analysis of intermixed human and mouse cells has, however, frequently relied on the need to separate the two cell populations prior to transcriptome analysis, which introduces confounding effects on gene expression.

**Methods:**

To circumvent this problem, we here describe a bioinformatics-based, genome-wide transcriptome analysis technique, which allows the human and mouse transcriptomes to be decoded from a mixed mouse and human cell population. The technique is based on a bioinformatic separation of the mouse and human transcriptomes from the initial mixed-species transcriptome resulting from sequencing an excised tumor/stroma specimen without prior cell sorting.

**Results:**

Under stringent separation criteria, i.e., with a read misassignment frequency of 0.2 %, we show that 99 % of the genes can successfully be assigned to be of mouse or human origin, both *in silico*, in cultured cells and *in vivo*. We use a new species-specific sequencing technology—referred to as S^3^ (“S-cube”)—to provide new insights into the Notch downstream response following Notch ligand-stimulation and to explore transcriptional changes following transplantation of two different breast cancer cell lines (luminal MCF7 and basal-type MDA-MB-231) into mammary fat pad tissue in mice of different immunological status. We find that MCF7 and MDA-MB-231 respond differently to fat pad xenografting and the stromal response to transplantation of MCF7 and MDA-MB-231 cells was also distinct.

**Conclusions:**

In conclusion, the data show that the S^3^ technology allows for faithful recording of transcriptomic changes when human and mouse cells are intermixed and that it can be applied to address a broad spectrum of research questions.

**Electronic supplementary material:**

The online version of this article (doi:10.1186/s13058-015-0616-x) contains supplementary material, which is available to authorized users.

## Introduction

Genome-wide transcriptomic analysis provides important insights into many cellular processes, including tumor development [1]. However, in many situations it would be useful to simultaneously decode the transcriptomes of two different interacting cell types; for example, when human tumor cells are xenografted into mice. To obtain the transcriptomic information from the transplanted cells and the surrounding stroma has, however, frequently required physical separation of the human and mouse cells (for example, by fluorescence-activated cell sorting (FACS)-based separation using human- and mouse-specific cell surface markers) and this extensive handling of the cells prior to transcriptome analysis is likely to affect the transcriptomic profiles. Alternatively, species-specific quantitative real-time PCR (qPCR) analysis or cross-species hybridization of microarrays have been attempted [2, 3], but neither approach allows for a large-scale probing of the transcriptomes from two species. It would therefore be useful to develop new alternative technologies that enable simultaneous high-throughput analysis of human and mouse transcriptomes without prior cell sorting.

In this report, we establish such a technology and address two research questions in which human–mouse cell mixing is experimentally used: analysis of Notch signaling and xenografting of human breast cancer cells into mammary fat pads in mice. Notch signaling is an evolutionarily highly conserved cell–cell signaling system where a signal-sending cell expresses transmembrane ligands, such as Delta-like 4 (DLL4), that bind to the transmembrane Notch receptor on a neighboring, signal-receiving cell [4]. Thus, canonical Notch signaling is often studied by activating Notch receptors through co-culture of ligand- and receptor-expressing cells [5]. Because of the intermixing of ligand- and receptor-expressing cells it has proven difficult to decode transcriptional events specifically occurring in the receptor-expressing cells. As deregulated Notch signaling is linked to breast cancer, it is important from a breast cancer perspective to better understand the transcriptomic effects of Notch signaling. High levels of expression of Notch ligands or receptors have been often correlated with poor prognosis [6, 7]. Gain-of-function Notch mutations have been observed in breast tumors [8] and the negative regulator Numb is frequently lost or inactivated [9, 10]. Overexpression of activated forms of Notch in mammary tissue in transgenic mice leads to breast tumor development [11], and engrafting of human breast tumor cells with different levels of Notch has revealed a role for Notch in tumor progression and cellular metabolism [12]. Notch signaling is also implicated in tumor–stroma interactions, metastasis and therapy resistance in breast cancer [13–16].

Xenografting to the mammary fat pad is frequently used to explore mammary stem and progenitor cell differentiation [17] and to gain insights into growth and metastasis of human breast cancer cell lines [18] and patient-derived tumors [19]. Xenografting also provides information about the stromal responses to the tumor cells [20], which is important as the tumor–stroma interplay has emerged as an interesting research area. It is increasingly acknowledged that the stroma is not a passive bystander in the tumor process, but that the interplay between tumor and stroma is important for tumor initiation, progression and metastasis [21, 22]. Xenografting can also shed light on the differences between different types of breast cancer cells. Breast cancer can be classified into at least five different types [23], including luminal and basal-type tumors. These two tumor types differ with regard to prognosis, but also in terms of how transplanted luminal and basal-type tumor cell lines behave following transplantation in mice. Transplantation of luminal cell lines, such as MCF7, result in tumor formation but not metastasis, whereas transplantation of the basal-type cell line MDA-MB-231 results in both tumor growth and metastasis [24].

In this study, we develop a new bioinformatics-based approach, which we refer to as S^3^ (S-cube; for species-specific sequencing) technology, which allows simultaneous analysis of mouse and human transcriptomes. We demonstrate that the S^3^ technology can identify 99 % of all genes to be of mouse or human origin at a low species misassignment frequency, and we use the technology to provide new insights into transcriptional effects of Notch signaling and tumor–stroma interactions in xenografts.

## Methods

### Cell lines and cell culture

MCF7 (ATCC®, HTB-22™) and MDA-MB-231 (ATCC®, HTB-26™) human breast adenocarcinoma cell lines, 3T3-L1 mouse embryonic fibroblast cell line (ATCC®, CL-173™), 293T human embryonic kidney cell line (ATCC®, CRL-3216™), and MCF7-EGFP [12, 25, 26] were cultured in complete medium, composed of Dulbecco's modified Eagle medium DMEM, high glucose, pyruvate (Gibco®, cat. no. 41966-029) supplemented with 10 % fetal bovine serum (Gibco®, cat. no. 10270-106) and 1 % penicillin-streptomycin (10,000 U/mL, Gibco®, cat. no. 15140-122) at 37 °C in a humidified 5 % CO_2_ atmosphere. Both MCF7 and MDA-MB-231 cells were cultured without estrogen in the medium.

### Plasmids

Pi2EGFP-hDLL4 [27] was generously donated by Prof. Dr. Manfred Gessler (Biocenter of the University of Würzburg, Germany). pCAGG-IRES-GFP and MH100-HSP-lacZ were kindly donated by Prof. Johan Ericson (Karolinska Institutet, Sweden). 12xCSL-Luc was a kind gift from Dr. Tasuku Honjo (Kyoto University, Japan) [28]. Fc-DLL4 was generously donated by Prof. Tom Kadesch (University of Pennsylvania School of Medicine, USA) [29].

### Cellular co-culture analysis

We seeded 8.5 × 10^4^ mouse 3T3-L1 cells per well in two 12-well plates in triplicate per sample group, and seeded 1.7 × 10^6^ human MDA-MB-231 cells in one 100 mm dish. Cells were allowed to settle, and were transfected the following day after the medium was changed to be antibiotic free. 3T3-L1 cells were transfected with Pi2EGFP-hDLL4 or green fluorescent protein (GFP) (4 μg/well), using Lipofectamine® 3000 (Invitrogen™, cat. no. L3000-015), according to the manufacturer’s instructions. Six hours after transfection, MDA-MB-231 cells were scraped and 8.5 × 10^4^ cells in 1 mL complete medium per well were added to both 12-well plates. We added 20 μM N-[N-(3,5-difluorophenacetyl)-L-alanyl]-S-phenylglycine t-butyl ester (DAPT; GSI-IX; 2 μL of 10 mM stock, Selleck Chemicals, cat. no. S2215) per well to one plate, and added dimethyl sulfoxide (DMSO; 2 μL, Sigma-Aldrich®, cat. no. D4540) per well to the other as control. Cells were co-cultured for 6 hours, then lysed in 350 μL per well Buffer RLT (QIAGEN, cat. no. 79216) with 1 % 2-Mercaptoethanol (Sigma-Aldrich®, cat. no. M3148) and stored at –80 °C until RNA extraction.

To measure Notch activity from the co-culture, a parallel assay was set up as above, with the following modification: MDA-MB-231 cells were co-transfected with 12xCSL-Luc (30 μg/dish) and MH100-HSP-lacZ (3.0 μg/dish). Cells were co-cultured for 18 hours, then lysed in 200 μL per well luciferase lysis buffer (10 mM Tris pH 7.9, 300 mM NaCl, 1.5 mM MgCl_2_, 0.65 % NP40), and stored at –20 °C until luciferase assay was performed. Notch activity in cells expressing the 12xCSL-Luc reporter construct was measured by a luciferase assay using the same reagents as described [25, 26].

### Activation of Notch signaling by immobilized ligand

We seeded 1.7 × 10^6^ MDA-MB-231 cells per dish in one 100 mm dish. The following day, two 12-well plates were coated with Fc control or Fc-DLL4, in triplicate per sample group, as previously described [30], with the following modifications: Protein G (Pierce®, cat. no. 21193) 50 μg/mL in Dulbecco's phosphate-buffered saline (DPBS) for 1 hour at room temperature, Fc-DLL4 conditioned medium (approximately 1 μg/mL) [31] or ChromPure Human IgG Fc fragment (Jackson ImmunoResearch Laboratories, Inc., cat. no. 009-000-008) 1 μg/mL in 0.1 % bovine serum albumin (BSA)-DPBS for 1 hour at room temperature. MDA-MB-231 cells were scraped and 8.5 × 10^4^ cells in 1 mL complete medium per well were added to both 12-well plates. We added 20 μM DAPT (2 μL of 10 mM stock) per well to one plate, and added DMSO (2 μL) per well to the other as control. Cells were co-cultured for 6 hours, then lysed in 350 μL per well Buffer RLT with 1 % 2-Mercaptoethanol, and stored at –80 °C until RNA extraction.

To measure the Notch activity by immobilized ligand activation, a parallel assay was set up as above, with the following modification: MDA-MB-231 cells were co-transfected with 12xCSL-Luc (30 μg/dish) and MH100-HSP-lacZ (3.0 μg/dish). Cells were seeded on coated plates for 18 hours, then lysed in 200 μL per well luciferase lysis buffer and stored at –20 °C until luciferase assay was performed. Luciferase activity was analyzed as described above.

### Tumor and mammary gland extraction

For MCF7 tumors, 2 × 10^6^ MCF7-EGFP cells were mixed with BD Matrigel™ Basement Membrane Matrix (BD Biosciences, cat. no. 356234) and injected into the left and right cleared fourth inguinal mammary fat pads of two female athymic Nude-Foxn1^nu^ mice (Harlan Laboratories). The tumors were allowed to grow for 8 weeks, supplemented with estrogen and progesterone pellets (Innovative Research of America) to support growth. Tumors were excised upon reaching a volume of 500 mm^3^, immersed in RNALater solution (Ambion®, cat. no. AM7020), and stored at –80 °C.

For MDA-MB-231 tumors, 2 × 10^6^ MDA-MB-231 cells in 50 μL phosphate-buffered saline (PBS) were injected into the left intact fourth inguinal mammary fat pad of four female Fox Chase SCID® Congenic-CB17/Icr-Prkdc^scid^/IcrIcoCrl mice (Charles River Laboratories, strain code 236). The tumors were considered to be established when the largest diameter exceeded 3 mm. The established tumors were excised after approximately 4–5 weeks at an average volume of 500 mm^3^ (tumor volume = π/6 × length × width^2^). Mice were injected with 2.5 % Avertin, heart-perfused with 10 mL PBS, and tumors resected. Tumors were snap-frozen by a dry ice/ethanol slurry, and stored at –80 °C. These tumors were obtained from mice in the control group of another experiment, and were treated with IgG antibody twice a week for a total of eight times.

As control, the left fourth mammary gland of a female NSG-NOD.Cg-Prkdc^scid^ Il2rg^tm1Wjl^/SzJ mouse (Jackson Laboratories, strain 005557) was cleared, dissected, immediately preserved in RNALater solution, and stored at –80 °C.

### Homogenization, RNA extraction and cDNA library preparation

Homogenization of MCF7 and MDA-MB-231 tumors was performed by the “Lysis and Homogenization: 10–100 mg Frozen or Fresh Fibrous Tissue, Mortar and Pestle Protocol” of the PureLink^TM^ RNA Mini Kit (Ambion®, cat. no. 12183-018A). Homogenization of the thawed mouse mammary gland was performed by mincing the tissue in 200 μL TRIzol® Reagent (Ambion®, cat. no. 15596-026) with a scalpel, using an RNase/DNase-free pestle and 1.5 mL tube to dissociate larger pieces in solution, and passing tissue solution through a 23-gauge needle ten times.

RNA extraction for all cell lysates from the immobilized ligand assay, two-species co-culture, MCF7 cells and MDA-MB-231 cells was performed using the RNeasy Mini Kit (QIAGEN, cat. no. 74104). RNA extraction of homogenized MCF7 and MDA-MB-231 tumors was performed using TRIzol Reagent followed by purification using the PureLink RNA Mini Kit. RNA extraction of the homogenized mammary gland was performed using TRIzol Reagent and PureLink® DNase (Ambion®, cat. no. 12185010) treatment. RNA concentration and RNA integrity number (RIN) were calculated by an Agilent 2100 Bioanalyzer system using the Agilent RNA 6000 Nano Kit (Agilent Technologies Inc., cat. no. 5067-1511).

cDNA libraries for all samples were created using the TruSeq® RNA Sample Prep Kit v2–48, Set B (Illumina®, cat. no. RS-122-2002), as per the TruSeq® RNA Sample Preparation v2 Guide, Low-Throughput Protocol. The following RNA amounts were used as starting material: 100 ng per sample of cell lysates, 200 ng of mammary gland, and 1 μg per sample of MCF7 and MDA-MB-231 tumors. Quantification and quality control of the cDNA libraries was performed using the Agilent DNA 1000 Kit (Agilent Technologies Inc., cat. no. 5067-1504). Quantification of pooled libraries was performed using the Qubit® 2.0 Fluorometer (Invitrogen™, cat. no. Q32866) and Qubit® dsDNA HS Assay Kit (Invitrogen™, cat. no. Q32851), and concentration of pooled libraries was diluted to 2 nM. All kits were used as per the manufacturer’s instructions.

### Alignment and analysis of technical performance

**Fig. 1:**
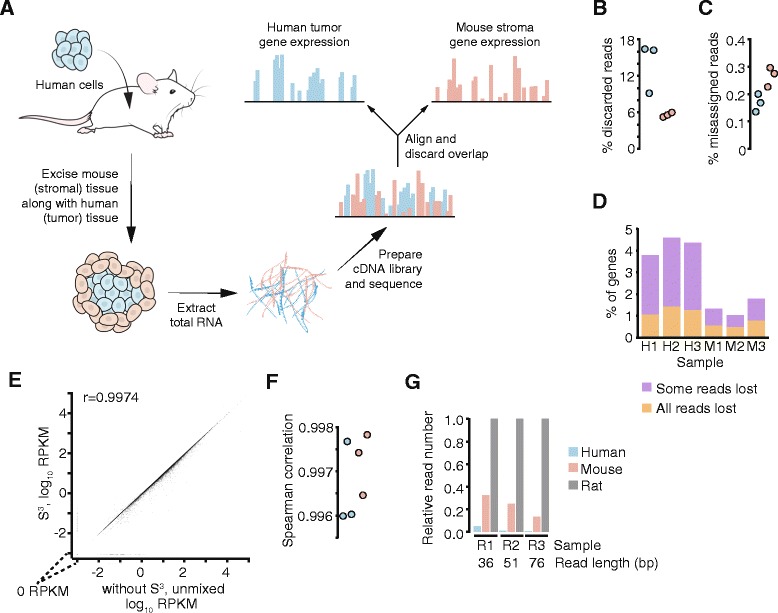
Species-specific sequencing—separation of mouse and human transcriptomes *in silico*. **a** Schematic flowchart of the principle steps in the S^3^ technology. A specimen of mixed human and mouse cells (for example, from a tumor–stroma xenograft experiment) is subjected to RNA-seq. The mixed transcriptome is bioinformatically separated into human and mouse transcriptomes, discarding transcripts with a defined maximum number of mismatches. **b** Fraction of reads that are species-ambiguous; i.e., cannot be assigned only to one species and therefore discarded. **c** Fraction of misassigned reads; i.e., species-specific reads that align to mouse genes for human samples or to human genes for mouse samples. Data in **b** and **c** are from three human (79 bp reads, in blue) and three mouse (51 bp reads, in pink) samples. **d** Percentage of expressed genes with full (orange) or partial (purple) loss of reads after S^3^ in the three human (H1–3) and mouse (M1–3) samples. **e** Scatterplot and Spearman correlation between a mouse sample (M1) and mouse expression values from S^3^ applied to a mix of human (H1) and mouse (M1) samples. **f** Spearman correlations for the human (blue) and mouse (pink) S^3^ components of *in silico* mixes of H1 + M1, H2 + M2 and H3 + M3. **g** The number of reads assigned by S^3^ as human, mouse or rat for three rat samples, normalized by the number of rat reads

The cDNA libraries were sequenced on a HiSeq 2000 with a 50–52 read length, single-end, for different samples. The reads were aligned to both the human genome (assembly hg19) and the mouse genome (assembly mm10) using STAR [32] with default settings (e.g., 10 mismatches maximum) in the version from 11 Feb 2013. For each species, we filtered for reads that had a unique match to that species' genome (quality score 255) and did not align to the other species' genome. For technical comparison in some analyses we also kept a set of aligned reads which mapped uniquely but without the filter for the other species genome. We also aligned reads using Bowtie [33] with the setting -best to both genome and transcriptome (from Ensembl for mouse, GENCODE for human), for comparison of alignment performance. We then calculated gene expression values using rpkmforgenes.py [34] (with the settings -rmnameoverlap -bothendsceil and RefSeq annotation downloaded 1 Jul 2014 from the UCSC genome browser). We removed microRNA and snoRNA genes since they had had extreme variability in previous projects, plus they are not normally polyadenylated, and applied TMM (trimmed mean of M-values) normalization of the RPKM values. Using only the reads mapping to the protein coding transcripts, we calculated the read losses due to ambiguous mapping as reads mapping to both species divided by reads mapping to the species of origin (Fig. 1b). The definition of “full loss” genes is the number of genes going from non-zero to zero expression upon discarding multi-species mapping reads, whereas the “partial loss” genes are those getting fewer reads but still non-zero. Since the true origin of each sequence was known, we could also calculate the number of misassigned reads (Fig. 1c). The samples in the in-silico analysis were three human samples from [35] (H1-3), three mouse samples (M1-3) [36], mbrNoAmp-s_1 (M4) and mbrNoAmp-s_2 (M5) [37] and the rat samples SRR1586060 (R1) [38], SRR1613356 (R2) [39] and ERR530739 (R3) [40]. Raw sequence reads have been deposited at the NCBI Sequence Read Archive (SRP056041) and processed gene expression values at the NCBI Gene Expression Omnibus (GSE66744). The detected gene cutoff was set to 1 read and the fold changes added a pseudo-value of 0.3 RPKM (based on the background value in Ramsköld et al. [41]) before calculating the ratio. For Xenome, we used the graft and host output files and aligned the reads with STAR using the default settings.

### Statistics and additional bioinformatics

**Fig. 2:**
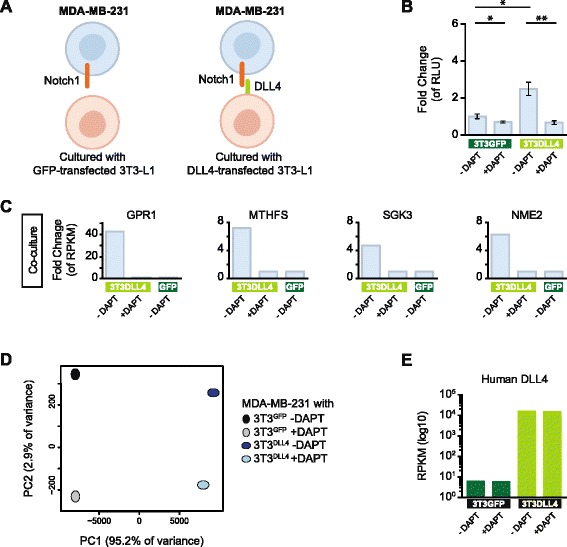
Analysis of ligand-induced Notch signaling using S^3^ technology. **a** Schematic depiction of the co-culture system used to analyze the Notch downstream response. The human MDA-MB-231 cells express robust levels of the Notch1 receptor and are co-cultured with mouse 3T3-L1 cells, which in some experiments are transfected with the Delta-like 4 (DLL4) ligand. **b** Analysis of 12xCSL-Luc activity for various combinations of co-culture of 3T3-L1 and MDA-MB-231 cells, where the latter are transfected with the Notch reporter 12xCSL-Luc. Note the increase in reporter activity where 3T3-L1 cells transfected with the DLL4 ligand are co-cultured with MDA-MB-231 cells, and that this increase is abrogated by the addition of N-[N-(3,5-difluorophenacetyl)-L-alanyl]-S-phenylglycine t-butyl ester (DAPT). Relative luciferase units (RLU) were normalized to beta-galactosidase values before fold change analysis. *p < 0.05, **p < 0.01 (Student’s *t*-test). Replicates per treatment group (*n* = 3) are from one culture split prior to transfection and measurement. **c** Fold change of expression levels (RPKM) for four genes (GPR1, MTHFS, SGK3 and NME2) from MDA-MB-231 cells co-cultured with 3T3-L1 cells transfected with DLL4 or green fluorescent protein (GFP) in the presence (+DAPT) or absence (-DAPT) of DAPT, as indicated. **d** Principal component analysis (PCA) of the genome-wide transcriptomes in MDA-MB-231 cells in response to DLL4 ligand-stimulation and DAPT treatment, as described in the figure. **e** Expression levels of human DLL4 in the co-cultures of MDA-MB-231 and 3T3-L1 cells, as described. Note the high level of DLL4 expression in cells transfected with a human DLL4 plasmid (the two bars to the right, light green)

**Fig. 3:**
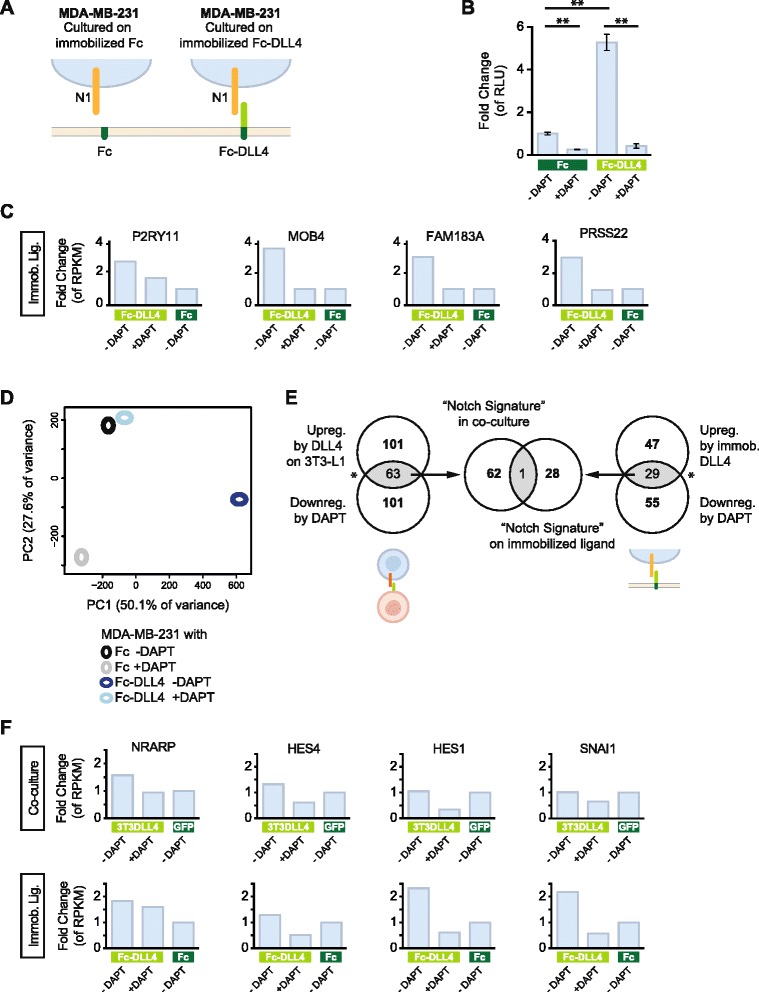
Analysis of two different modes of Notch ligand presentation. **a** Schematic depiction of activation of Notch by immobilized ligand (Fc-DLL4) or with Fc as control. **b** Analysis of 12xCSL-Luc activity in MDA-MB-231 cells cultured on immobilized Fc-DLL4 or Fc alone as control, and in the presence or absence of N-[N-(3,5-difluorophenacetyl)-L-alanyl]-S-phenylglycine t-butyl ester (DAPT), as indicated. Note the increase in reporter activity when cells are cultured on Delta-like 4 (DLL4) and that this activity is abrogated by the addition of DAPT. Relative luciferase units (RLU) were normalized to beta-galactosidase values before fold change analysis. *p < 0.05, **p < 0.01 (Student’s *t*-test). Replicates per treatment group (*n* = 3) are from one culture split prior to transfection and measurement. **c** Fold change of expression levels (RPKM) of four genes (P2RY11, MOB4, FAM183A and PRSS22) in the MDA-MB-231 cells in response to DLL4 ligand-stimulation and DAPT treatment, as described in the figure. **d** Principal component analysis (PCA) of the genome-wide transcriptomes in MDA-MB-231 cells in response to DLL4 ligand-stimulation and DAPT treatment, as indicated. **e** Comparison of Notch response signatures derived by DLL4 presented from co-cultured cells (*left*) or immobilized DLL4 (*right*). In the upper left circle are the 164 genes that are >2-fold upregulated in MDA-MB-231 cells by DLL4 on 3T3-L1 cells and the lower left circle denotes the 164 genes that are downregulated by DAPT. The overlap between these two categories (63 genes) are genes that are both upregulated by DLL4 and downregulated by DAPT, i.e. the Notch signature. For the immobilized ligand there are 76 genes that are upregulated by immobilized DLL4, and 84 genes are downregulated by DAPT. The overlap is 29 genes, which are both upregulated by immobilized DLL4 and downregulated by DAPT. Comparison of the “co-culture” and “immobilized” signatures identifies only one gene in common (the middle section of the figure). *p < 0.05 (Fisher’s exact test). **f** Fold change of expression levels (RPKM) from four well-established Notch target genes (NRARP, HES4, HES1 and SNAI1) in the MDA-MB-231 cells in response to DLL4 ligand-stimulation by co-culture (*upper row*) or DLL4 immobilized ligand (immob. lig., *lower row*), respectively, and in the presence or absence of DAPT as indicated. *GFP* green fluorescent protein

For the figures showing principal component analysis, we used the prcomp function in R. We used DAVID functional annotation tool for a Gene Ontology enrichment analysis [42, 43], taking one term from each cluster in the output and requiring a 5 % Benjamini-adjusted *p*-value produced by DAVID. Because a maximum of 3000 genes can be analyzed using the official gene symbol identifier, a random number generator was used to choose 3000 genes from lists containing greater than 3000 genes. For differential expression testing we reused a test from Ramsköld et al. [37], but modified to take RPKM values as input instead of exon inclusion frequency. This test used the same statistic as in significance analysis of microarrays (SAM), and then *p*-values were calculated by permuting the samples to create a null distribution, and produced false discovery rates (FDRs) using the Benjamini-Hochberg method. The complete script for the S^3^ technology (from fastq.gz file input to final human/mouse gene expression quantification output) is given in [44]. Fisher’s exact test was deployed to test gene list overlap, using 24,112 as the total number of human genes and 23,225 as the total number of mouse genes, and the values are presented in Additional file 1 (Table S1). The *p*-value tables from SAM (see above) are listed in Additional file 2 (Table S2). Luciferase assay comparisons in Figs. 2b and 3b were analyzed using two-tailed unpaired Student’s *t*-test with Welch’s correction. The genes corresponding to the gene counts in figure panels of *in vitro* comparisons (Fig. 3e; Additional file 3: Figure S4 and Additional file 4: Table S3) are listed in Additional file 5 (Table S4).

### Statement of ethical approval

Animal experiments were conducted in accordance with the institutional animal care policies of Karolinska Institutet, University of Turku and Åbo Akademi University. Stockholms Norra Djurförsöksetiska granted ethical permit number N151/14. The Finnish animal ethics committee granted ethical permit numbers STH471A/ESLH-2008-05395/Ym-23 7.7 2009, STH169A/ESLH-2009-01942/Ym-23 11.3 2009, and ESLH-2008-05395/Ym-23 23.6 2011.

## Results

### Species-specific sequencing—separation of mouse and human transcriptomes *in silico*

We wanted to establish a technology that faithfully reports the human and mouse transcriptomes from an intermixed human/mouse tissue sample. The gist of the technology is to align all sequence reads and discard the overlaps and a flowchart for the principle steps in the S^3^ technology is presented in Fig. 1a, with a program-by-program presentation available as Additional file 3 (Figure S1). We began our analysis by first exploring to what extent transcriptomes of mouse and human origin could be separated *in silico*. We set up a pipeline where RNA-seq data were aligned to both the mouse and human genomes by the program STAR, removing reads that aligned to both. We have optimized the settings for this pipeline (Additional file 6: Table S5), finding that the settings -Mu 1 (max. 1 mismatch for unique) -Ms 1 (max. 1 mismatch for shared) -D 2 (max. 2 mismatch difference) gave the best balance between sensitivity and specificity. The code is available at [44]. We tested this pipeline on three human (79 bp reads) and three mouse (51 bp reads) samples and found that, on average, 9.8 % of the reads were discarded as species-ambiguous (Fig. 1b) and that, on average, 0.2 % of the reads were assigned to the wrong species (Fig. 1c). The discarded reads corresponded to a full loss of, on average, 0.9 % of the expressed genes (all reads lost) and a partial loss of 1.9 % of the expressed genes (some reads lost) (Fig. 1d). The misassigned reads were spread over approximately 2500 genes on average; i.e., they contribute to a low background level of expression, although genes for nuclear proteins tended to have more such reads than other genes (Additional file 7: Table S6A). To ascertain that species misassignment and potential biases in read loss does not cause S^3^ expression values to lose accuracy, we mixed pairs of human and mouse samples *in silico* and compared expression values before and after mixing the samples and using S^3^ (Fig. 1e,f), with good correlation. As recent reports have described related technologies to decode mixed-genome transcriptomes [45, 46–48], we directly compared our method to one of these methods, Xenome, for which a detailed script is available [45]. The comparison revealed that the S^3^ technology has 1.6 ± 0.2 (SEM) times better separation between species and retains 4.8 ± 0.5 times more reads as compared to Xenome combined with STAR alignment (Additional file 3: Figure S2).

We next asked whether the S^3^ technology would be able to cope with a comparison of transcriptomes from more than two species, which would be useful for example when human tumor cells and rat cancer-associated fibroblasts (CAFs) are xenografted into the mouse to study specific aspects of tumor–CAF interaction. When we aligned rat samples to three genomes—mouse, human and rat—and separated the reads by species with S^3^, we noticed large numbers of reads being assigned as mouse-specific (Fig. 1g). The same was not true for mouse samples (Additional file 3: Figure S3), and we suspect the difficulty lies with gaps and/or incorrect sequence in the rat genome assembly, which currently has a much lower quality than the mouse and human genome assemblies. In conclusion, the *in silico* analysis provides proof-of-concept that species-specific differences can be effectively used to dichotomize a mixed-species transcriptome, and the technology can also be extended to analyze more than two species, in particular where high-quality genome information is available.

### Analysis of ligand-induced Notch signaling using S^3^ technology

We used the S^3^ technology to gain further insights into Notch signaling and, more specifically, to assess whether the mode of Notch ligand presentation to Notch receptor-expressing cells affects the Notch downstream response. Notch signaling starts when a Notch ligand (such as DLL4) on one cell interacts with a Notch receptor on a neighboring cell leading to γ-secretase complex-mediated proteolytic processing of the Notch receptor, ultimately releasing its intracellular domain (Notch ICD). Notch ICD then translocates to the nucleus, where it binds to the DNA-binding protein CSL (RBP-Jκ) to activate transcription of downstream genes (for reviews see [4, 49]). To study receptor activation, there are two principally different ways to activate Notch signaling by ligand: the ligand can be expressed on a neighboring cell or immobilized in the culture dish. To what extent these two ways of providing ligand yields different responses is not known, as it has been difficult to establish a genome-wide transcriptome in the Notch receptor cells in the co-culture situation because of confounding effects of the ligand-presenting cells.

To induce Notch signaling we co-cultured mouse embryonic fibroblast 3T3-L1 cells transiently transfected with human DLL4 plasmid (3T3-L1^DLL4^) or GFP plasmid (3T3-L1^GFP^) as control, with human breast adenocarcinoma MDA-MB-231 cells, which express robust levels of the Notch1 receptor [31, 50] (schematically depicted in Fig. 2a). Activation of Notch signaling after ligand stimulation was verified by upregulation of the transfected Notch reporter 12xCSL-Luc in the MDA-MB-231 cells after 18 hours of co-culture with the DLL4-expressing cells, and this activation was abrogated by the γ-secretase inhibitor DAPT (Fig. 2b). Analysis of the transcriptome of all combinations of the 3T3-L1 and MDA-MB-231 cells revealed that more than 98 % of expressed genes could be distinguished in both human and mouse transcriptomes (Additional file 8: Table S7, % remaining genes), in keeping with the *in silico* sorting results presented above. The proportion of human and mouse sequence reads was close to 50 % (Additional file 8: Table S7C, % reads), indicating that there were approximately equal numbers of MDA-MB-231 and 3T3-L1 cells in each co-culture experiment. In our single-species samples, we observed that 0.1–0.3 % reads were assigned to the wrong species (Additional file 8: Table S7, % reads), due to sequencing read errors, which normally occur at this frequency in RNA-Seq [51, 52]. We next compared the MDA-MB-231 transcriptomes derived after exposure to 3T3-L1^DLL4^ or 3T3-L1^GFP^ cells. Co-culturing with the 3T3-L1^DLL4^ cells upregulated 164 genes and downregulated 415 genes as compared to co-culturing with the 3T3-L1^GFP^ cells (Additional file 3: Figure S4A).

We next analyzed the effect of blocking Notch signaling by the γ-secretase inhibitor DAPT, which abrogates Notch receptor cleavage [49]. Addition of 20 μM DAPT at the onset of the co-culturing (i.e., for a total of 6 hours) resulted in downregulation of 63 of the 164 genes that were upregulated by co-culture with DLL4-expressing cells (Additional file 3: Figure S4A). A number of genes, such as GPR1, MTHFS, SGK3 and NME2, showed very strong DAPT-mediated abrogation of the DLL4-induced activation (Fig. 2c) but, at the genome-wide transcriptome level, DAPT only caused a partial response, as visualized in the principal component analysis (PCA) in Fig. 2d. This may be a consequence of the DAPT treatment being too short to completely eradicate Notch signaling (e.g., some Notch1 ICD present at the start of the 6-hour DAPT blockade could linger on and still exert a gene-inducing effect after 6 hours). The transcriptome data showed very high expression of human DLL4 in the 3T3-L1^DLL4^ cells in both the absence and presence of DAPT (Fig. 2e), indicating that transfection of DLL4 was successful and that DLL4 expression was not affected by γ-secretase inhibition. The whole-genome gene expression quality control (QC) data, demonstrating good sequencing depth saturation, are presented in Additional file 3 (Figure S5) and the whole-genome gene expression QC density plots are shown in Additional file 3 (Figure S6).

To compare the data from DLL4 presented on neighboring cells with a Notch transcriptome resulting from immobilized DLL4 ligand, we cultured MDA-MB-231 cells on Fc-DLL4 ligand for 6 hours (schematically depicted in Fig. 3a). The 12xCSL-Luc reporter construct was, as expected, robustly activated by immobilized DLL4, as compared to exposure to Fc fragment alone (Fig. 3b). Culturing on Fc-DLL4 ligand upregulated 76 genes, whereas 127 genes were downregulated (Additional file 3: Figure S4B). Of the 76 upregulated genes, 29 were downregulated by DAPT treatment for 6 hours (Additional file 3: Figure S4B). As for the induction of Notch signaling by DLL4 in co-culture, a number of genes, such as P2RY11, MOB4, FAM183A and PRSS22, showed robust upregulation by DLL4 and an almost complete downregulation by DAPT (Fig. 3c) but, at the genome-wide level, the DAPT blockade of the immobilized DLL4 was incomplete, although more effective than in the co-culture setting (PCA; Fig. 3d).

To compare the “co-culture” and “immobilized” DLL4 transcriptomes we first decoded a Notch transcriptomic signature in both settings, as defined by all genes that were upregulated by DLL4 ligand and where the upregulation was reduced by DAPT treatment (Fig. 3e); 63 genes met these criteria in the “co-culture” setting, whereas 29 genes were upregulated by immobilized DLL4 ligand and downregulated by DAPT. Interestingly, when these two gene sets were cross-compared, only one gene was common to both the “co-culture” and “immobilized” Notch signatures (Fig. 3e; Additional file 4: Table S3A-B, PCA Additional file 3: Figure S7A). This indicates that the mode of ligand presentation is important for the transcriptional response. A comparison of the genes activated in MDA-MB-231 cells by co-culturing with 3T3-L1^GFP^ cells or cultured alone on Fc using Gene Ontology yielded several genes associated by an inflammatory and cytokine response that were upregulated only by co-culturing (Additional file 7: Table S6B, Additional file 4: Table S3C, PCA Additional file 3: Figure S7B). In line with differential responses to the mode of ligand presentation, the transcriptional responses for the Notch target genes NRARP, HES4, HES1 and SNAI1 [30, 53] were to some extent different in the “co-culture” or “immobilized” settings (Fig. 3f).

Finally, analysis of the transcriptomic changes in the ligand-presenting cells (i.e., the 3T3-L1 cells) revealed that 245 genes were uniquely upregulated and 371 genes downregulated in the DLL4-expressing cells as compared to GFP-expressing 3T3-L1 cells (Additional file 3: Figure S4C, PCA Additional file 3: Figure S7C). Of the 245 genes, 98 were sensitive to DAPT treatment (Additional file 3: Figure S4C). Overall, these data show that the S^3^ technology can be used to faithfully report mouse and human transcriptomes from co-cultured cells, and also provide novel insights into the Notch downstream response with regard to different modes of ligand presentation.

### Transcriptional consequences of xenografting luminal and basal-type breast tumor cell lines in mice

Xenografting tumor cells into mammary tissue in mice has yielded important information about breast tumor growth *in vivo* [24, 54, 55], but it has been difficult to simultaneously obtain a genome-wide understanding of transcriptome changes in both tumor and stroma. We therefore decided to use the S^3^ technology to address: 1) how the transcriptomes of luminal (MCF7) and basal-type (MDA-MB-231) transcriptomes change upon transplantation to mammary tissue in mice; and 2) how the surrounding stroma is affected by transplantation of the two cell types. We also used different modes of transplantation: MDA-MB-231 cells were transplanted directly into the mammary fat pad whereas MCF7 cells were grafted into cleared fat pads. The immune status of the recipient mice was likewise different: nude mice were used for the MCF7 transplantations, whereas SCID mice were used for MDA-MB-231 transplantations. MCF7 or MDA-MB-231 cells were transplanted and the human (tumor) and mouse (stroma) transcriptomes from excised combined tumor and stroma were bioinformatically separated (see Fig. 1a). The percentage of human sequence reads in the analyzed mixed-species samples of xenografted MCF7 cell/stroma specimens ranged from approximately 25 to 75 %, while xenografted MDA-MB-231 cell/stroma specimens had approximately 80–90 % human reads (Additional file 8: Table S7G), reflecting that we had dissected out a balanced proportion of tumor and stroma (one sample, Mouse1R_MCF7-EGFP that contained less than 5 % tumor or stroma contribution, was discarded and not used for further analysis). The whole-genome gene expression QC data demonstrate that there is good sequencing depth saturation (Additional file 3: Figure S5). The whole-genome gene expression QC density plots are shown in Additional file 3 (Figure S6). For the MCF7 cells, 2142 genes were upregulated and 1564 genes were downregulated in the xenograft situation as compared to *in vitro* cultured cells (Additional file 9: Table S8A). The xenograft and *in vitro* cultured MCF7 transcriptomes clustered separately as judged by PCA, and the spread among the xenograft samples was larger (Additional file 3: Figure S7D), probably reflecting a larger individual variation in the tumor situation. Of note, the MCF7 cells were not grown with estradiol supplementation *in vitro* to mimic the estrogen and progesterone pellets utilized for the xenografting, but genes involved in estrogen signaling do not look to be over-represented in the MCF7 tumor samples compared to MCF7 cells (Additional file 3: Figure S8) [56, 57, 58].

**Fig. 4:**
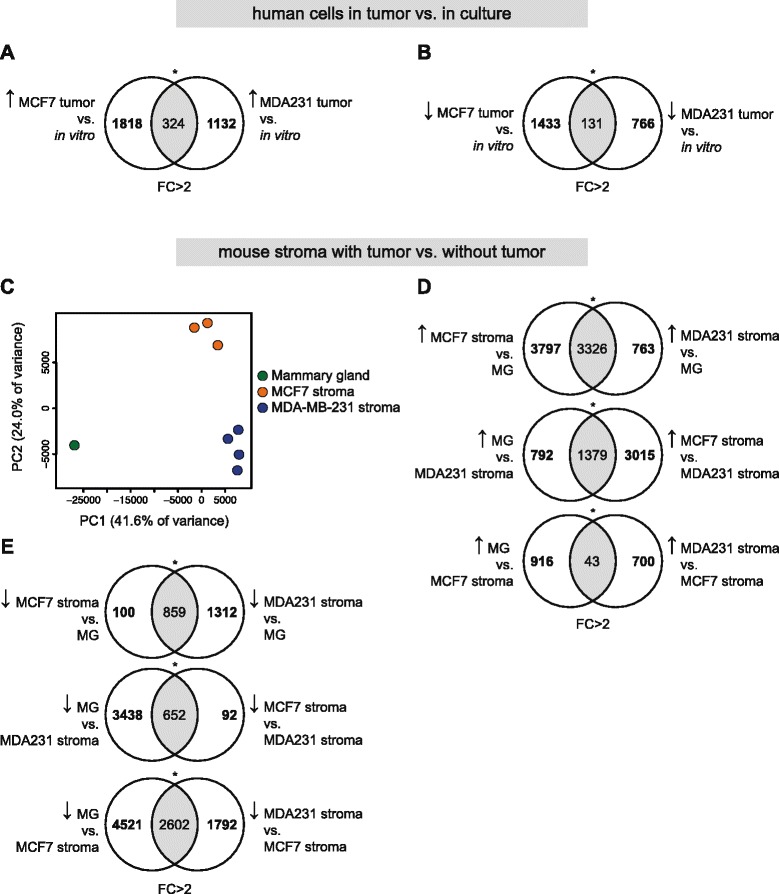
Transcriptional consequences of xenografting MCF7 and MDA-MB-231 cells into mammary fat pads in mice. **a,b** Venn diagrams comparing MCF7 and MDA-MB-231 transcriptomes *in vitro* and *in vivo*. **a** The overlap (324 genes) between the 2142 genes that are upregulated in MCF7 tumors vs. *in vitro* culturing (*left*) and the 1456 genes that are upregulated in MDA-MB-231 tumors vs. *in vitro* culturing (*right*). **b** Similar analysis as in **a**, but for genes downregulated in MCF7 and MDA-MB-231 tumors as compared to *in vitro* culturing. **c** Principal component analysis (PCA) of untransplanted mammary gland, MCF7 tumor stroma (top to bottom: 3R, 1L, 3L) and MDA-MB-231 tumor stroma (top to bottom: 1, 32, 13, 22). **d** The number of genes upregulated (fold change (FC) >2) in MCF7 tumor stroma compared to MDA-MB-231 tumor stroma, as a subset of the number of genes upregulated in MCF7 or MDA-MB-231 tumor stroma compared to untransplanted mammary gland. **e** The number of genes downregulated (FC >2) in MCF7 tumor stroma compared to MDA-MB-231 tumor stroma, as a subset of the number of genes downregulated in MCF7 or MDA-MB-231 tumor stroma compared to untransplanted mammary gland. *p < 0.05 (Fisher’s exact test)

For the basal-type MDA-MB-231 cells, 1456 genes were upregulated and 897 genes downregulated in the xenograft compared to the *in vitro* culturing (Additional file 9: Table S8A). Gene Ontology analysis demonstrated that genes upregulated in xenografted MDA-MB-231 showed an enrichment of genes involved in cell adhesion (Additional file 7: Table S6C). PCA also revealed that the MDA-MB-231 transcriptomes from *in vitro* culturing clearly segregated from the xenograft transcriptomes (Additional file 3: Figure S7E). A comparison of the 2142 and 1456 genes upregulated in MCF7 and MDA-MB-231 cells after xenografting, respectively, showed that only 324 genes were upregulated in both cell types (Fig. 4a) and 131 genes were downregulated (Fig. 4b), suggesting distinctly different adaptation needs for the two cell lines upon being xenografted. An analysis cut-off of fold-change (>2-fold), rather than FDR (<5 %), was used since sample numbers between comparison groups were not similar (Additional file 9: Table S8).

On the stromal side, a large number of genes were up- or downregulated in the stroma when MCF7 or MDA-MB-231 cells were transplanted, as compared to untransplanted mammary gland (Additional file 9: Table S5B). For all comparisons presented in Table S8, scatterplots (Additional file 3: Figure S9) show the differentially expressed genes are not lowly expressed, indicating that the cutoff of 0.3 RPKM successfully removed “noisy” genes from the analysis. When the MCF7 stromal transcriptome was directly compared to the stroma surrounding MDA-MB-231 cells we identified 4394 genes upregulated in the MCF7 versus the MDA-MB-231 stroma, whereas 743 genes were downregulated (Additional file 9: Table S8B). Using only *in vivo* data, PCA clustered the individual MCF7 and MDA-MB-231 stroma experiments separately and they were also well separated from the untransplanted mammary gland transcriptome, with MCF7 clustering roughly midway (considering the greater importance of PC1 over PC2) between the untransplanted experiment and the MDA-MB-231 stroma (Fig. 4c). This predicts a smaller gene overlap between MDA-MB-231 stroma and untransplanted mammary gland than between MCF7 stroma and untransplanted mammary gland; this was indeed observed when comparing the overlap among upregulated (>2-fold) or downregulated genes (>2-fold) against the third group (Fig. 4d-e), especially among upregulated genes (Fig. 4d). Gene Ontology analysis of the stromal transcriptomes revealed that the MDA-MB-231-induced stromal transcriptome was over-represented with regard to genes involved in blood vessel morphogenesis (Additional file 7: Table S6D). In the MCF7-induced stromal gene set, genes involved in chromatin modification were found (Additional file 7: Table S6D). An unsupervised hierarchical clustering of all human and mouse transcriptomes from both the *in vivo* and *in vitro* analysis and the different transcriptomes showed the expected clustering (Additional file 3: Figure S10). Finally, we investigated the expression of a published list of universal stromal markers [59], and could confirm that it indeed reflected the stromal response to a tumor, for both the MCF7 and the MDA-MB-231 xenografts (Additional file 3: Figure S11).

## Discussion

In this report, we describe a bioinformatics-based approach to simultaneously decode the transcriptomes of mixed-species samples without tissue handling and physical separation of the cells. The sorting of the mRNAs to human or mouse origin is based on sequence differences in the coding regions and, under stringent conditions (i.e., with a read misassignment frequency of 0.2 %), on average 99 % of all genes could be assigned as either mouse or human, both *in silico*, *in vitro* and *in vivo*. Four recent studies describe similar approaches to bioinformatically sort human and mouse transcriptomes [45, 46–48], but with some noticeable differences in how mismatches are used. In particular, S^3^ makes use of the difference in the number of mismatches for a read. A direct comparison with the Xenome strategy [45], which like the S^3^ technology provides a user-friendly code, revealed that the S^3^ technology has better species separation and better read retention. Although all these studies have focused on the separation of mouse and human transcriptomes, the technology can in principle be applied to more than two species, and we provide an example from a comparison of human, mouse and rat transcriptomes, although the yet somewhat incomplete characterization of the rat genome likely reduced the specificity. Furthermore, the degree to which the two mRNA populations can be bioinformatically separated will depend on the extent of evolutionary distance between the two species but the strategy can in fact be used to provide important information also from very closely related genomes. Recently an intercross between *Mus musculus domesticus* and *Mus musculus castaneus* was used to unravel the transcriptional profiles in preimplantation mouse development, and the difference between these two mouse subspecies was sufficient to decode the transcriptional contribution from the maternal and paternal genomes [60].

We used the S^3^ technology to explore whether the mode of ligand presentation affected the Notch downstream response. While a synthetic Notch reporter construct and a number of classical Notch target genes, such as Nrarp, Hes1 and Snail, were activated by both immobilized ligand and ligand presented on co-cultured cells, the genome-wide transcriptional profiles were quite distinct. In fact, only one gene was identified that in both settings was upregulated by DLL4-ligand and the ligand-mediated upregulation abrogated by DAPT. The reasons for this difference between the “co-culture” and “immobilized” Notch signatures remains to be further explored. The short time window for activation (i.e., 6 hours of ligand stimulation) may lead to differences if genes have different temporal activation profiles in the two settings. Furthermore, as the PCA data indicate, the 6-hour DAPT treatment may be insufficient to eradicate all Notch signaling. It is also possible that in the co-culture situation production of secreted factors from the ligand-expressing cell may synergize with Notch ligand activation to govern gene expression, which may at least in part explain the difference between the “co-culture” and “immobilized” Notch signatures. In summary, as both immobilized ligand stimulation and presentation of ligand from juxtaposed cells in co-culture are methods frequently used to activate Notch signaling (for example in breast cancer research) it may be important to keep in mind that the two modes of ligand presentation may yield partly different responses.

The importance of the interaction between tumor and stroma is increasingly recognized [21, 22] and we show that the S^3^ technology can be productively used to decode the tumor and stroma transcriptomes from mammary fat pad xenograft experiments. Our data demonstrate that both the MCF7 and MDA-MB-231 cells undergo widespread transcriptional changes upon transplantation to the mammary fat pad, and that the changes in MDA-MB-231 cells were more profound. This shows that growth in the *in vivo* environment of the mammary gland has a considerable impact on the tumor cell transcriptomes and that the response is cell line-specific. The Gene Ontology analysis demonstrated that the gene categories representing extracellular matrix components were enriched among the genes upregulated in xenografted MCF7 cells, while the cell adhesion category was enriched in the MDA-MB-231 cells following transplantation. The difference in responses in MCF7 and MDA-MB-231 cells is likely not only affected by which cell line was transplanted but also by the fact that different transplantation techniques were used for the two cell types: MDA-MB-231 cells were transplanted directly into the mammary fat pad whereas MCF7 cells were grafted into cleared fat pads. Furthermore, the immune status of the recipient mice was different: the mice used for MCF7 transplantation were nude mice, which lack T cells but retain B cells and natural killer (NK) cells, whereas xenografting of MDA-MB-231 cells was done in SCID mice, which have no T cells and B cells but retain NK cells. While it is plausible that both the origin of the transplanted cells, the transplantation technique and the immune status of the mice contribute to the profound differences in tumor transcriptomes, the precise impact of each of these parameters can be explored by the S^3^ technology in further studies.

The stromal response to xenografting MCF7 or MDA-MB-231 cells was also remarkably different, with nearly 4000 genes upregulated in stroma from MCF7 transplants as compared to MDA-MB-231 transplants. In the PCA, the genome-wide transcriptome from MCF7 stroma was intermediate between the MDA-MB-231 and the untransplanted stromal transcriptomes. This is in line with the fact that the basal-type MDA-MB-231 cell line is more aggressive and also produces metastases upon transplantation [59], but as discussed above the difference in xenografting techniques and immune status of the mice used for transplanting the two cell types is likely to substantially contribute to the differences in the response. It is interesting to note that genes upregulated in both the MCF7 and MDA-MB-231 surrounding stroma include genes involved in cytokine response as well as several supposedly universal stromal markers [59], whereas the MCF7 stroma also was enriched for genes involved in chromatin remodeling.

## Conclusions

In conclusion, our data from co-cultured cells and xenograft experiments show that the S^3^ technology allows for faithful reporting of transcriptomic changes that will provide new insights from experiments based on mixed-species cell interactions *in vitro* or *in vivo*.

## Additional files

Additional file 1: Table S1. Fisher’s exact test for count data (R). (XLSX 10 kb) Additional file 2: Table S2.
*p*-values and FDR from SAMlike_test_on_rpkms.py. (XLSX 14232 kb) Additional file 3: Figure S1. Pipeline of data processing. **Figure S2.** Comparison with other methods to separate human and mouse reads, both in terms of sensitivity (A) and specificity (B). **Figure S3.** Comparison of three species (rat, mouse, human) separation of rat (R1–R3) and mouse (M4, M5) RNA-seq samples, similar to Fig. 1g but with absolute number of reads on the y axis. **Figure S4.** Comparison of the number of genes up- and downregulated in (A) MDA-MB-231 cells co-cultured with 3T3-L1 cells expressing DLL4 or GFP, (B) MDA-MB-231 cells on immobilized Fc-DLL4 or Fc, and (C) 3T3-L1 cells, expressing DLL4 or GFP, co-cultured with MDA-MB-231 cells. **Figure S5.** Whole-genome gene expression QC: Depth Saturation. **Figure S6.** Whole-Genome Gene Expression QC: Density Plots after TMM Normalization. **Figure S7.** Additional principal component analyses (PCA). **Figure S8.** Scatterplot of genes in Estrogen-related signaling, differentially expressed in MCF7 cells *in vitro* and MCF7 cells in tumor. **Figure S9.** Scatterplots of Table S8 comparison groups. **Figure S10.** Hierarchical Clustering. **Figure S11.** FINAK_BREAST_CANCER_SDPP_SIGNATURE: Scatterplot of genes in the stroma-derived prognostic predictor of breast cancer disease outcome (Finak et al. 2008 [59]), differentially expressed in (A) Mammary Gland (MG) compared to MCF7 tumor stroma, (B) MG compared to MDA-MB-231 tumor stroma, and MCF7 tumor stroma compared to MDA-MB-231 tumor stroma. (PDF 11548 kb) Additional file 4: Table S3. Fold change for analysis of Notch activation by co-culture and immobilized ligand. (PDF 410 kb) Additional file 5: Table S4. Gene lists from *in vitro* comparisons. (XLSX 53 kb) Additional file 6: Table S5. Optimization of S^3^ parameters. (XLS 65 kb) Additional file 7: Table S6. Gene Ontology. (XLSX 270 kb) Additional file 8: Table S7. Alignment Statistics. (PDF 657 kb) Additional file 9: Table S8. Fold change and false discovery rate for analysis of gene expression in tumor and stroma. (PDF 426 kb)

## Abbreviations

BSA: Bovine serum albumin
CAF: Cancer-associated fibroblast
DAPT: N-[N-(3,5-difluorophenacetyl)-L-alanyl]-S-phenylglycine t-butyl ester
DLL4: Delta-like 4
DMEM: Dulbecco's modified Eagle medium
DMSO: Dimethyl sulfoxide
DPBS: Dulbecco's phosphate-buffered saline
FACS: Fluorescence-activated cell sorting
FDR: False discovery rate
GFP: Green fluorescent protein
HSP: Heat shock protein
ICD: Intracellular domain
IRES: Internal ribosome entry site
Luc: Luciferase
NK: Natural killer
PBS: Phosphate-buffered saline
PCA: Principal component analysis
QC: Quality control
qPCR: Quantitative real-time polymerase chain reaction
RPKM: Reads per kilobase of transcript per million reads mapped
SAM: Significance analysis of microarrays
SEM: Standard error of the mean
Seq: Sequencing

## References

[1] Ozsolak F, Milos PM (2011). RNA sequencing: advances, challenges and opportunities. Nat Rev Genet..

[2] Farren M, Weston S, Brown H, Broadbent N, Powell S, Shaw R (2012). Expression of stromal genes associated with the angiogenic response are not differentiated between human tumour xenografts with divergent vascular morphologies. Angiogenesis..

[3] Park ES, Kim S-J, Kim SW, Yoon S-L, Leem S-H, Kim S-B (2011). Cross-species hybridization of microarrays for studying tumor transcriptome of brain metastasis. Proc Natl Acad Sci..

[4] Andersson ER, Sandberg R, Lendahl U (2011). Notch signaling: simplicity in design, versatility in function. Development..

[5] Chapman G, Liu L, Sahlgren C, Dahlqvist C, Lendahl U (2006). High levels of Notch signaling down-regulate Numb and Numblike. J Cell Biol..

[6] Reedijk M, Odorcic S, Chang L, Zhang H, Miller N, McCready DR (2005). High-level coexpression of JAG1 and NOTCH1 is observed in human breast cancer and is associated with poor overall survival. Cancer Res..

[7] Speiser J, Foreman K, Drinka E, Godellas C, Perez C, Salhadar A (2012). Notch-1 and Notch-4 biomarker expression in triple-negative breast cancer. Int J Surg Pathol..

[8] Robinson DR, Kalyana-Sundaram S, Wu Y-M, Shankar S, Cao X, Ateeq B (2011). Functionally recurrent rearrangements of the MAST kinase and Notch gene families in breast cancer. Nat Med..

[9] Pece S, Serresi M, Santolini E, Capra M, Hulleman E, Galimberti V (2004). Loss of negative regulation by Numb over Notch is relevant to human breast carcinogenesis. J Cell Biol..

[10] Meurette O, Stylianou S, Rock R, Collu GM, Gilmore AP, Brennan K (2009). Notch activation induces Akt signaling via an autocrine loop to prevent apoptosis in breast epithelial cells. Cancer Res..

[11] Jhappan C, Gallahan D, Stahle C, Chu E, Smith GH, Merlino G (1992). Expression of an activated Notch-related int-3 transgene interferes with cell differentiation and induces neoplastic transformation in mammary and salivary glands. Genes Dev..

[12] Landor SK-J, Mutvei AP, Mamaeva V, Jin S, Busk M, Borra R (2011). Hypo- and hyperactivated Notch signaling induce a glycolytic switch through distinct mechanisms. Proc Natl Acad Sci U S A..

[13] Boelens MC, Wu TJ, Nabet BY, Xu B, Qiu Y, Yoon T (2014). Exosome transfer from stromal to breast cancer cells regulates therapy resistance pathways. Cell..

[14] Shimoda M, Principe S, Jackson HW, Luga V, Fang H, Molyneux SD (2014). Loss of the Timp gene family is sufficient for the acquisition of the CAF-like cell state. Nat Cell Biol..

[15] Xing F, Kobayashi A, Okuda H, Watabe M, Pai SK, Pandey PR (2013). Reactive astrocytes promote the metastatic growth of breast cancer stem-like cells by activating Notch signalling in brain. EMBO Mol Med..

[16] Martz CA, Ottina KA, Singleton KR, Jasper JS, Wardell SE, Peraza-Penton A (2014). Systematic identification of signaling pathways with potential to confer anticancer drug resistance. Sci Signal..

[17] Deome KB, Faulkin LJ, Bern HA, Blair PB (1959). Development of mammary tumors from hyperplastic alveolar nodules transplanted into gland-free mammary fat pads of female C3H mice. Cancer Res..

[18] Minn AJ, Gupta GP, Siegel PM, Bos PD, Shu W, Giri DD (2005). Genes that mediate breast cancer metastasis to lung. Nature..

[19] Zhang X, Claerhout S, Prat A, Dobrolecki LE, Petrovic I, Lai Q (2013). A renewable tissue resource of phenotypically stable, biologically and ethnically diverse, patient-derived human breast cancer xenograft models. Cancer Res..

[20] Unger C, Kramer N, Walzl A, Scherzer M, Hengstschläger M, Dolznig H (2014). Modeling human carcinomas: physiologically relevant 3D models to improve anti-cancer drug development. Adv Drug Deliv Rev..

[21] Hanahan D, Weinberg RA (2011). Hallmarks of cancer: the next generation. Cell..

[22] Pietras K, Östman A (2010). Hallmarks of cancer: interactions with the tumor stroma. Exp Cell Res..

[23] Prat A, Perou CM (2011). Deconstructing the molecular portraits of breast cancer. Mol Oncol..

[24] Holliday DL, Speirs V (2011). Choosing the right cell line for breast cancer research. Breast Cancer Res..

[25] Gustafsson MV, Zheng X, Pereira T, Gradin K, Jin S, Lundkvist J (2005). Hypoxia requires notch signaling to maintain the undifferentiated cell state. Dev Cell..

[26] Hansson EM, Lanner F, Das D, Mutvei A, Marklund U, Ericson J (2010). Control of Notch-ligand endocytosis by ligand-receptor interaction. J Cell Sci..

[27] Diez H, Fischer A, Winkler A, Hu CJ, Hatzopoulos AK, Breier G (2007). Hypoxia-mediated activation of Dll4-Notch-Hey2 signaling in endothelial progenitor cells and adoption of arterial cell fate. Exp Cell Res..

[28] Kato H, Taniguchi Y, Kurooka H, Minoguchi S, Sakai T, Nomura-Okazaki S (1997). Involvement of RBP-J in biological functions of mouse Notch1 and its derivatives. Development..

[29] Buas MF, Kabak S, Kadesch T (2009). Inhibition of myogenesis by notch: evidence for multiple pathways. J Cell Physiol..

[30] Sahlgren C, Gustafsson MV, Jin S, Poellinger L, Lendahl U (2008). Notch signaling mediates hypoxia-induced tumor cell migration and invasion. Proc Natl Acad Sci U S A..

[31] Jin S, Mutvei AP, Chivukula IV, Andersson ER, Ramsköld D, Sandberg R (2013). Non-canonical Notch signaling activates IL-6/JAK/STAT signaling in breast tumor cells and is controlled by p53 and IKKα/IKKβ. Oncogene..

[32] Dobin A, Davis CA, Schlesinger F, Drenkow J, Zaleski C, Jha S (2013). STAR: ultrafast universal RNA-seq aligner. Bioinformatics..

[33] Langmead B, Trapnell C, Pop M, Salzberg SL (2009). Ultrafast and memory-efficient alignment of short DNA sequences to the human genome. Genome Biol..

[34] Ramsköld D, Storvall H, Sandberg R. Sandberg Lab RNA-Sequencing. http://sandberg.cmb.ki.se/rnaseq/. Accessed 12 Jun 2015.

[35] Tsoi LC, Iyer MK, Stuart PE, Swindell WR, Gudjonsson JE, Tejasvi T (2015). Analysis of long non-coding RNAs highlights tissue-specific expression patterns and epigenetic profiles in normal and psoriatic skin. Genome Biol..

[36] Nassar D, Latil M, Boeckx B, Lambrechts D, Blanpain C. Genomic landscape of carcinogen induced mouse skin squamous cell carcinoma. EMBL-EBI ArrayExpress: E-MTAB-2889. http://www.ebi.ac.uk/arrayexpress/experiments/E-MTAB-2889/. Accessed 12 Jun 2015.

[37] Ramsköld D, Luo S, Wang Y-C, Li R, Deng Q, Faridani OR (2012). Full-length mRNA-Seq from single-cell levels of RNA and individual circulating tumor cells. Nat Biotechnol..

[38] Zhou Z, Karlsson C, Liang T, Xiong W, Kimura M, Tapocik JD (2013). Loss of metabotropic glutamate receptor 2 escalates alcohol consumption. Proc Natl Acad Sci U S A..

[39] Aguirre A, Montserrat N, Zacchigna S, Nivet E, Hishida T, Krause MN (2014). *In vivo* activation of a conserved MicroRNA program induces mammalian heart regeneration. Cell Stem Cell..

[40] Heyne HO, Lautenschläger S, Nelson R, Besnier F (2014). Genetic influences on brain gene expression in rats selected for tameness and aggression. Genetics..

[41] Ramsköld D, Wang ET, Burge CB, Sandberg R (2009). An abundance of ubiquitously expressed genes revealed by tissue transcriptome sequence data. PLoS Comput Biol..

[42] Huang DW, Sherman BT, Lempicki RA (2009). Systematic and integrative analysis of large gene lists using DAVID bioinformatics resources. Nat Protoc..

[43] Huang DW, Sherman BT, Lempicki RA (2009). Bioinformatics enrichment tools: paths toward the comprehensive functional analysis of large gene lists. Nucleic Acids Res..

[44] S^3^ Technology Script. https://github.com/danielramskold/S3_species-specific_sequencing.

[45] Conway T, Wazny J, Bromage A, Tymms M, Sooraj D, Williams ED (2012). Xenome-a tool for classifying reads from xenograft samples. Bioinformatics..

[46] Bradford JR, Farren M, Powell SJ, Runswick S, Weston SL, Brown H (2013). RNA-Seq differentiates tumour and host mRNA expression changes induced by treatment of human tumour xenografts with the VEGFR tyrosine kinase inhibitor cediranib. PLoS One..

[47] Raskatov JA, Nickols NG, Hargrove AE, Marinov GK, Wold B, Dervan PB (2012). Gene expression changes in a tumor xenograft by a pyrrole-imidazole polyamide. Proc Natl Acad Sci U S A..

[48] Rossello FJ, Tothill RW, Britt K, Marini KD, Falzon J, Thomas DM (2013). Next-generation sequence analysis of cancer xenograft models. PLoS One..

[49] Andersson ER, Lendahl U (2014). Therapeutic modulation of Notch signalling—are we there yet?. Nat Rev Drug Discov..

[50] Kulic I, Robertson G, Chang L, Baker JHE, Lockwood WW, Mok W (2015). Loss of the Notch effector RBPJ promotes tumorigenesis. J Exp Med..

[51] Grabherr MG, Haas BJ, Yassour M, Levin JZ, Thompson DA, Amit I (2011). Full-length transcriptome assembly from RNA-Seq data without a reference genome. Nat Biotechnol..

[52] Mortazavi A, Williams BA, McCue K, Schaeffer L, Wold B (2008). Mapping and quantifying mammalian transcriptomes by RNA-Seq. Nat Methods..

[53] Lamar E, Deblandre G, Wettstein D, Gawantka V, Pollet N, Niehrs C (2001). Nrarp is a novel intracellular component of the Notch signaling pathway. Genes Dev..

[54] Mollard S, Mousseau Y, Baaj Y, Richard L, Cook-Moreau J, Monteil J (2011). How can grafted breast cancer models be optimized?. Cancer Biol Ther..

[55] Norum JH, Andersen K, Sørlie T (2014). Lessons learned from the intrinsic subtypes of breast cancer in the quest for precision therapy. Br J Surg..

[56] Marino M, Galluzzo P, Ascenzi P (2006). Estrogen signaling multiple pathways to impact gene transcription. Curr Genomics..

[57] Heldring N, Pike A, Andersson S, Matthews J, Cheng G, Treuter E (2007). Estrogen receptors : how do they signal and what are their targets. Physiol Rev..

[58] Tocris Bioscience. Estrogen Signaling Pathway. 2015. http://www.tocris.com/pathways/estrogenPathway.php#.VTZRkq2qpBc. Accessed 12 Jun 2015.

[59] Finak G, Bertos N, Pepin F, Sadekova S, Souleimanova M, Zhao H (2008). Stromal gene expression predicts clinical outcome in breast cancer. Nat Med..

[60] Deng Q, Ramsköld D, Reinius B, Sandberg R (2014). Single-cell RNA-seq reveals dynamic, random monoallelic gene expression in mammalian cells. Science..

