# Supplementary material for: Decoding breast cancer tissue–stroma interactions using species-specific sequencing
Source: Breast Cancer Res. 2015 Aug 13;17(1):109. doi: 10.1186/s13058-015-0616-x (PMC4534116; doi:10.1186/s13058-015-0616-x)
Supplement: Additional file 3: Figure S1. — Pipeline of data processing. Figure S2. Comparison with other methods to separate human and mouse reads, both in terms of sensitivity (A) and specificity (B). Figure S3. Comparison of three species (rat, mouse, human) separation of rat (R1–R3) and mouse (M4, M5) RNA-seq samples, similar to Fig. 1g but with absolute number of reads on the y axis. Figure S4. Comparison of the number of genes up- and downregulated in (A) MDA-MB-231 cells co-cultured with 3T3-L1 cells expressing DLL4 or GFP, (B) MDA-MB-231 cells on immobilized Fc-DLL4 or Fc, and (C) 3T3-L1 cells, expressing DLL4 or GFP, co-cultured with MDA-MB-231 cells. Figure S5. Whole-genome gene expression QC: Depth Saturation. Figure S6. Whole-Genome Gene Expression QC: Density Plots after TMM Normalization. Figure S7. Additional principal component analyses (PCA). Figure S8. Scatterplot of genes in Estrogen-related signaling, differentially expressed in MCF7 cells in vitro and MCF7 cells in tumor. Figure S9. Scatterplots of Table S8 comparison groups. Figure S10. Hierarchical Clustering. Figure S11. FINAK_BREAST_CANCER_SDPP_SIGNATURE: Scatterplot of genes in the stroma-derived prognostic predictor of breast cancer disease outcome (Finak et al. 2008 [59]), differentially expressed in (A) Mammary Gland (MG) compared to MCF7 tumor stroma, (B) MG compared to MDA-MB-231 tumor stroma, and MCF7 tumor stroma compared to MDA-MB-231 tumor stroma. (PDF 11548 kb) [file 13058_2015_616_MOESM3_ESM.pdf]

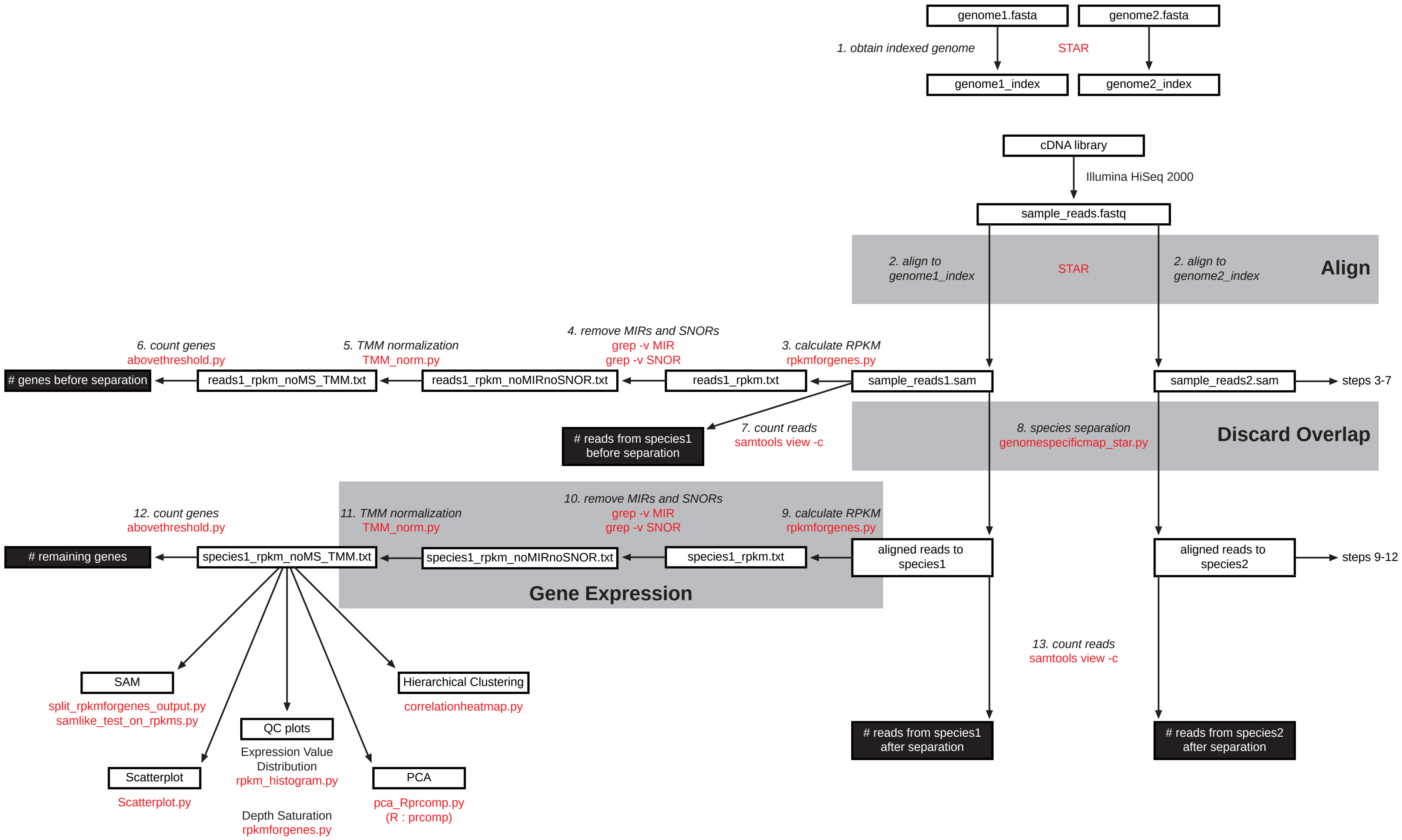

Figure S1. Pipeline of data processing.
This figure shows the steps used to produce the expression values (steps 1,2,8-11) as well as how to calculate numbers (black boxes) that are referenced in the formulas in Table S3. The grey boxes highlight the steps after cDNA library preparation and sequencing, indicated in Figure 1A.

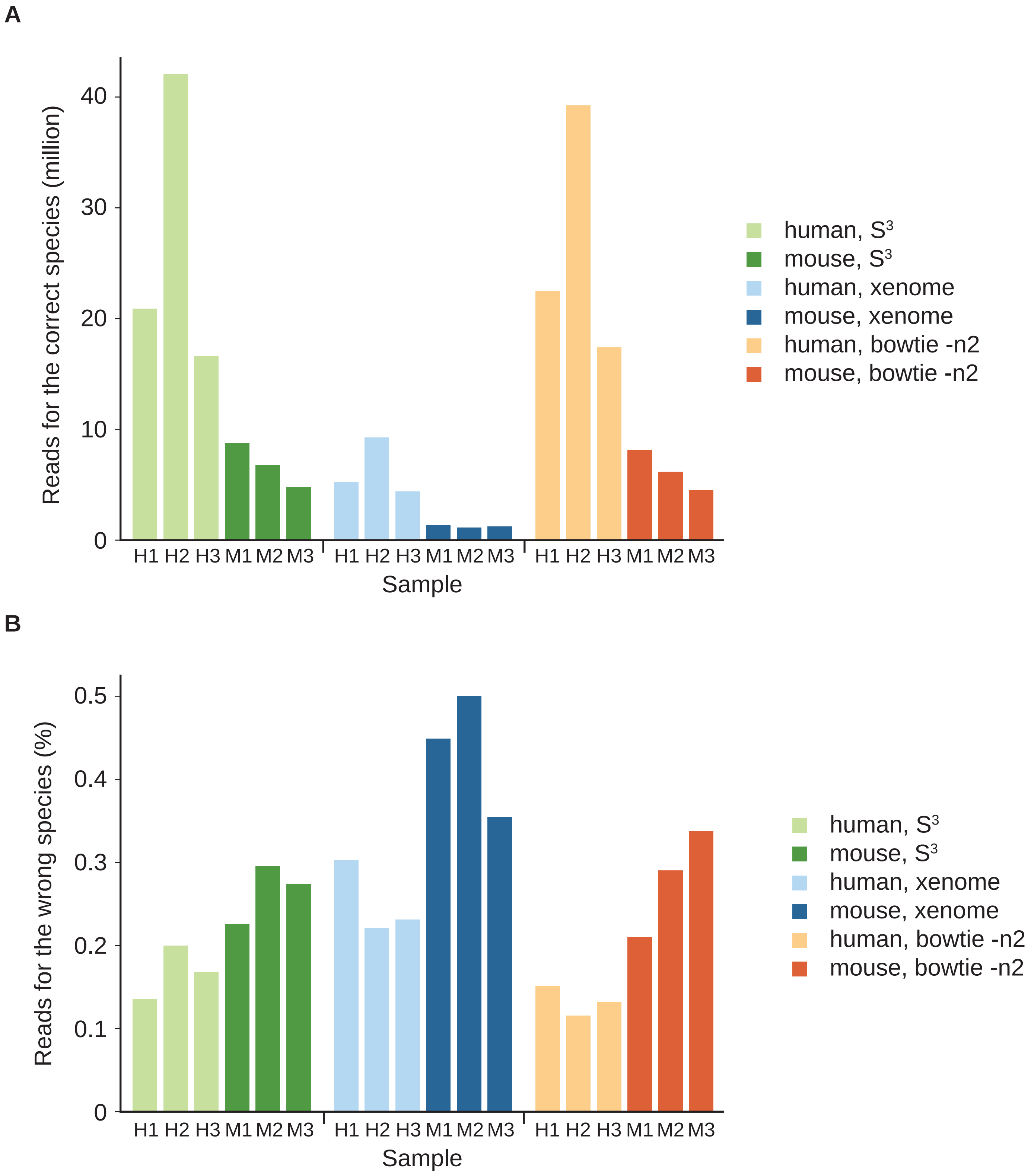

Figure S2. Comparison with other methods to separate human and mouse reads, both in terms of sensitivity (A) and specificity (B). We compared STAR-based S^3 to another algorithm of ours (genome+transcriptome bowtie alignment allowing two mismatches in the seed region) and to the species separation method Xenome (Conway et al. 2012), followed by STAR alignment with default parameters. M1-3 are mouse samples, H1-3 are human samples.

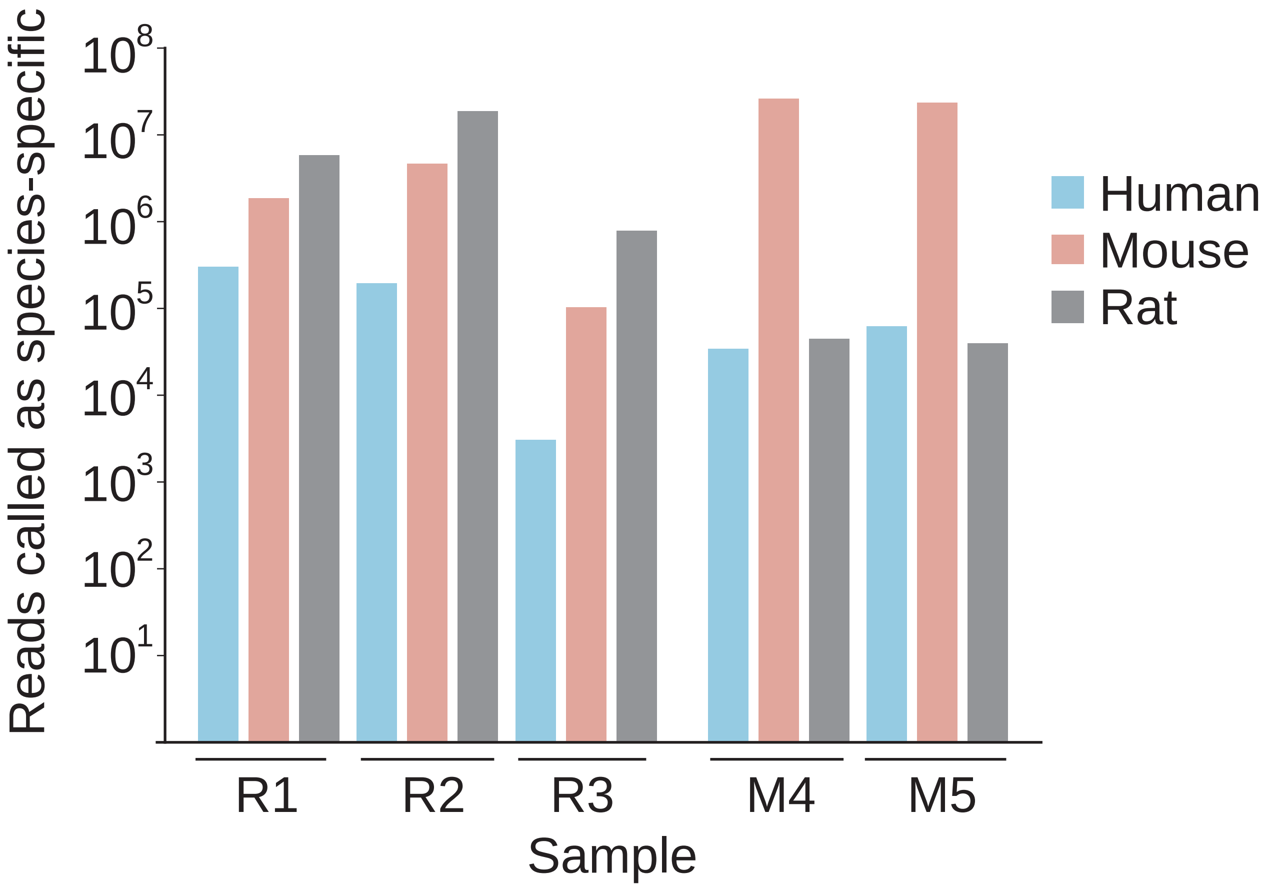

Figure S3. Comparison of three species (rat, mouse, human) separation of rat (R1-R3) and mouse (M4, M5) RNA-seq samples, similar to Figure 1F but with absolute number of reads on the y axis. The mouse samples have a 50 bp read length, similar to the 51 bp of R2.

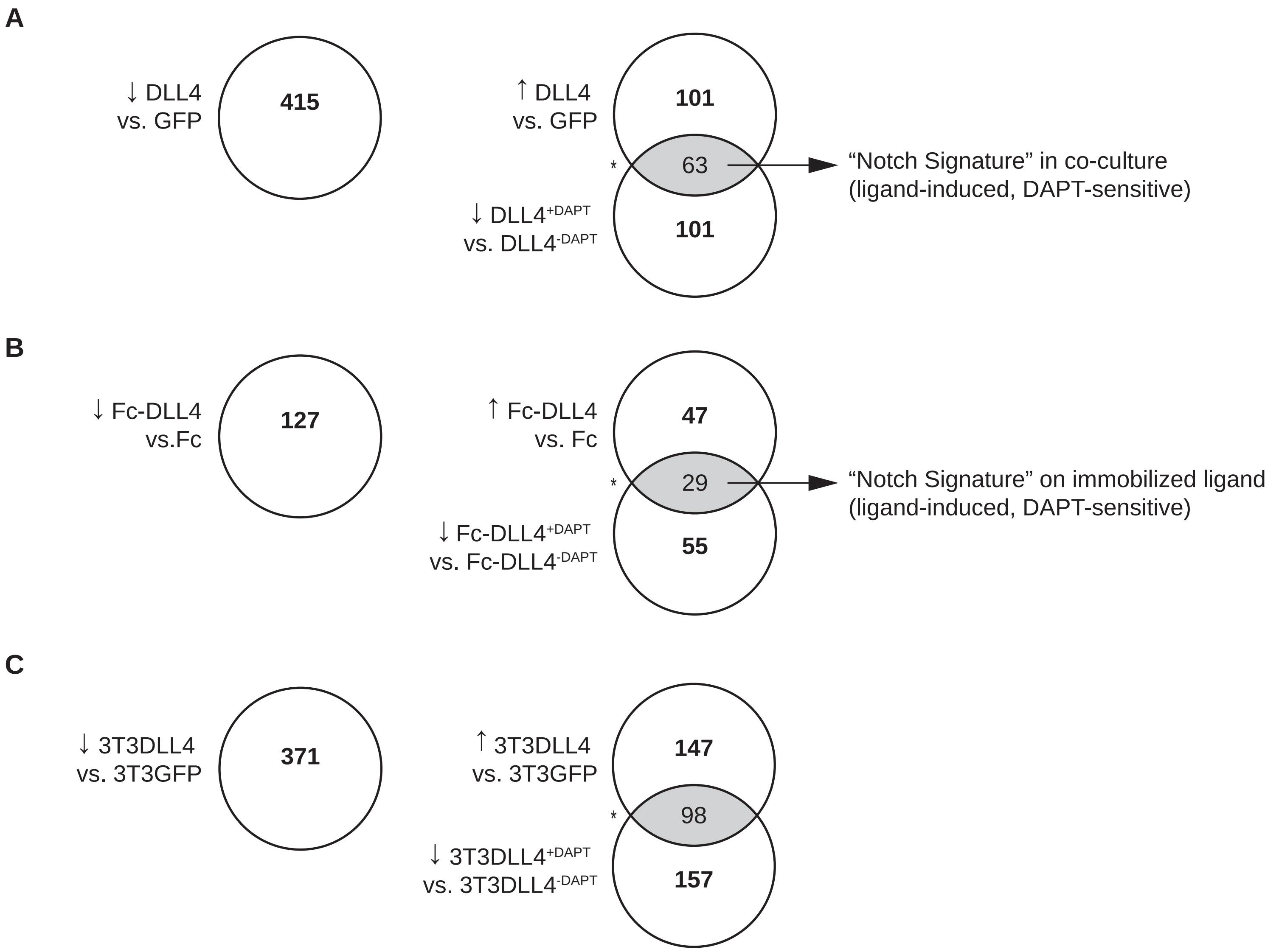

Figure S4. Comparison of the number of genes up- and downregulated in (A) MDA-MB-231 cells co-cultured with 3T3-L1 cells expressing DLL4 or GFP, (B) MDA-MB-231 cells on immobilized Fc-DLL4 or Fc, and (C) 3T3-L1 cells, expressing DLL4 or GFP, co-cultured with MDA-MB-231 cells. In (A) and (B), a “Notch Signature” is defined as genes upregulated through ligand induction and downregulated after DAPT treatment. Up- and downregulation is defined as FC>2. * signifies $p<0.05$ (Fisher’s exact test).

Human expression values:

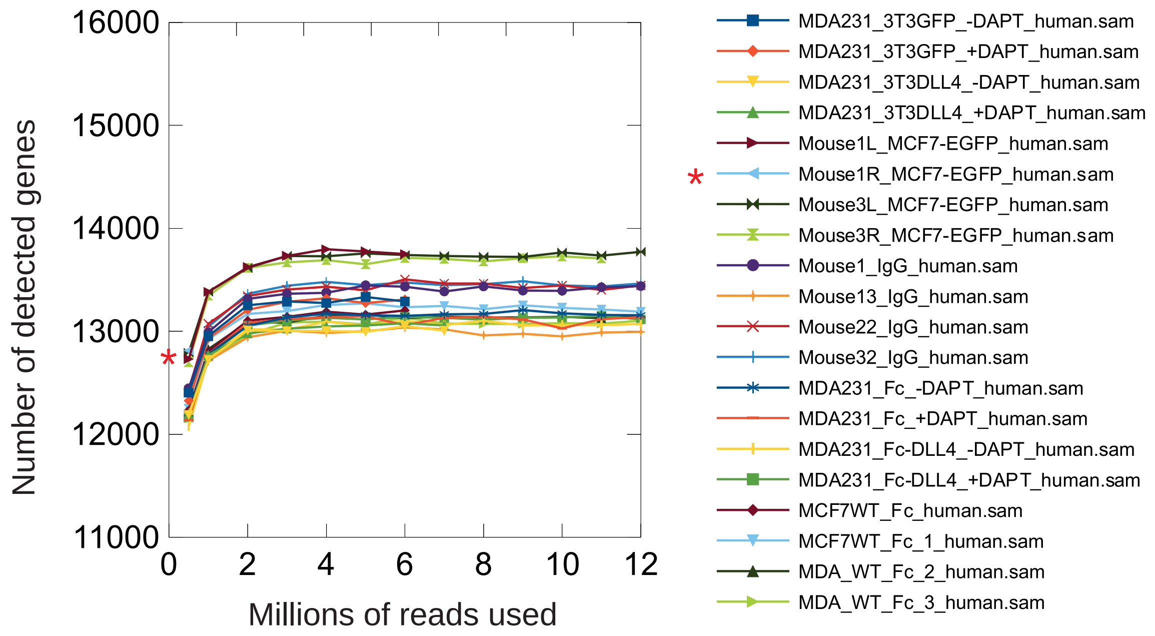

Mouse expression values:

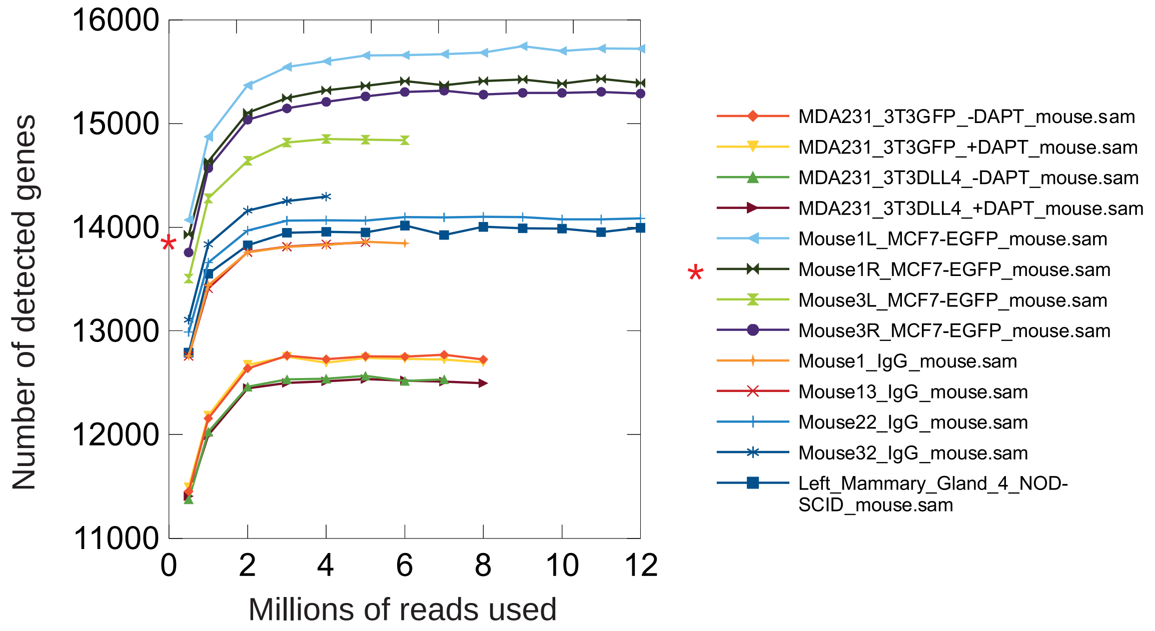

Figure S5. Whole-genome gene expression QC: Depth Saturation.
The graphs show the number of expressed genes (>0.3 RPKM) after downsampling to 0.5-12 million mapped reads. All samples generated in the study are shown, including one sample marked by a red asterisk which was excluded for failing quality control. The total number of reads for each sample is listed under “# reads” in Additional file 4: Table S3. Samples containing cell lines yielded fewer detected genes than samples containing mouse stroma, presumably because the latter is a more complex tissue containing many cell types. As seen above, gene detection levels out after 2-3 million reads, which is less then our sequencing depths (excluding the sample marked by *).

in vitro (human):

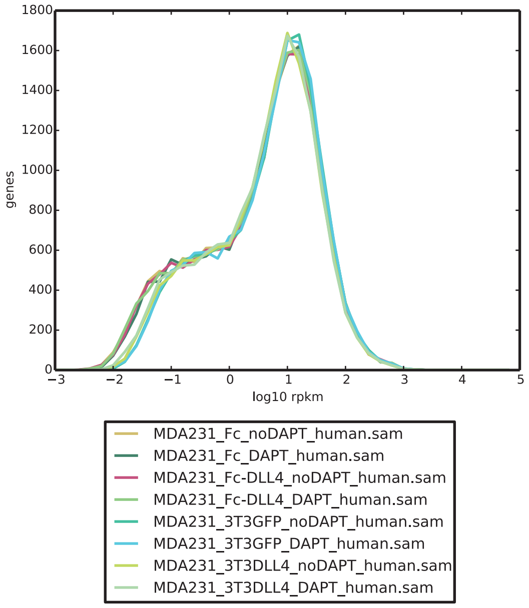

in vitro (mouse):

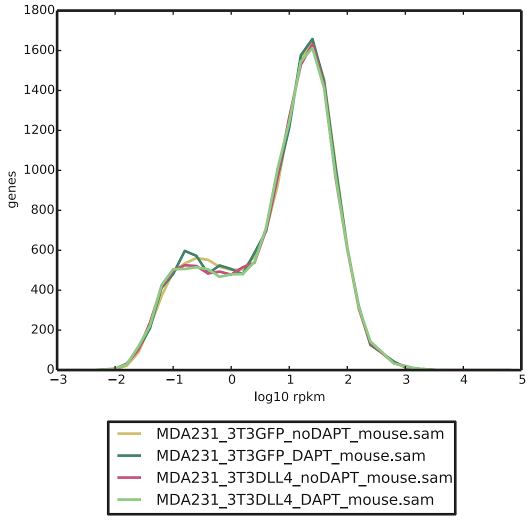

in vivo (human):

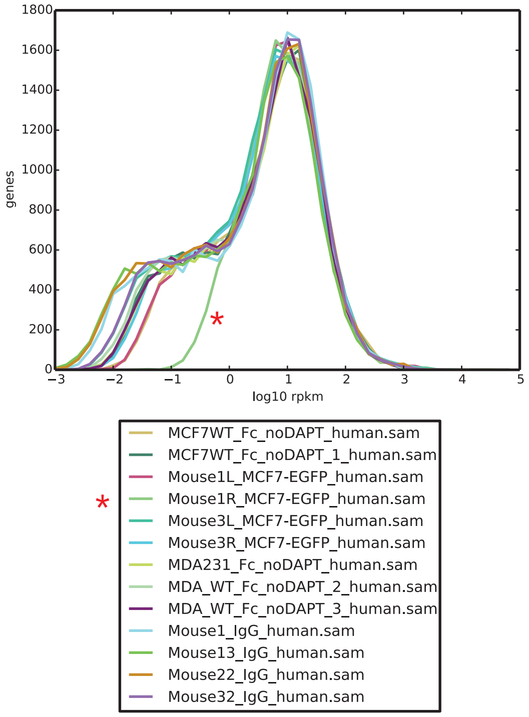

in vivo (mouse):

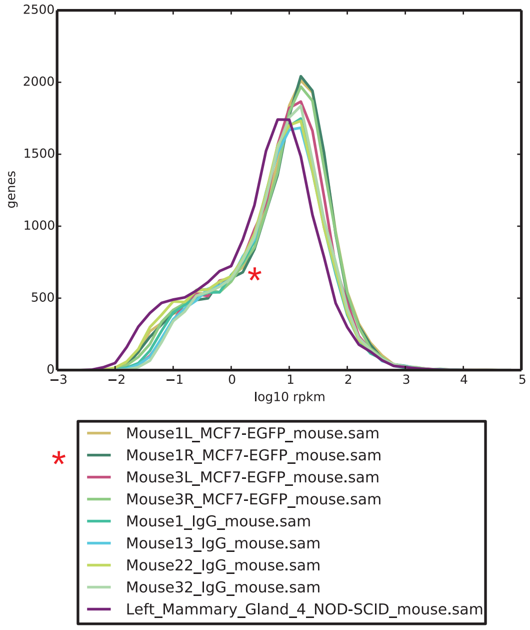

Figure S6. Whole-Genome Gene Expression QC: Density Plots after TMM Normalization. The plots show RPKM distributions for all samples generated in the study, including one sample marked by a red asterisk which was excluded for failing quality control (see also Figure S5).

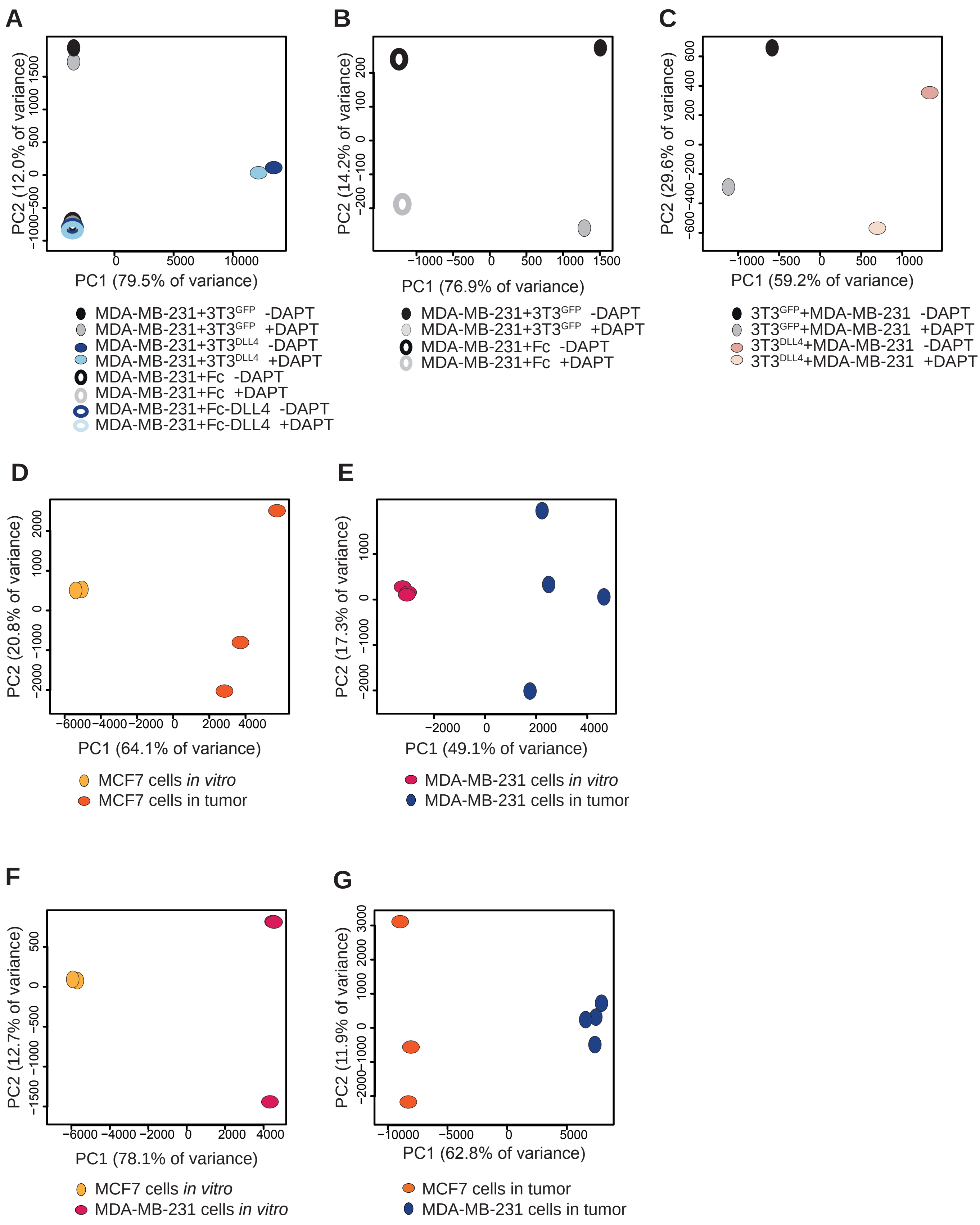

Figure S7: Additional principal component analyses (PCA). These use the expression values of all human or mouse genes and show the first and second principal components. Additional sample information can be found in Additional File 4: Table S3.

(A) MDA-MB-231 (DLL4-ligand-activated) cells cultured with 3T3-L1 cells expressing/not expressing DLL4 or on immobilized ligand and in the presence or absence of DAPT, as indicated.
(B) MDA-MB-231 cells cultured with 3T3-L1 cells or on Fc-fragment.
(C) 3T3-L1 (ligand-expressing) cells in co-culture.
(D) MCF7 cells *in vitro* (left to right: Fc_1, Fc) and in tumor (top to bottom: 1L, 3L, 3R).
(E) MDA-MB-231 cells *in vitro* (top to bottom: Fc, Fc_2, Fc_3) and in tumor (top to bottom: 22, 32, 13, 1).
(F) MCF7 (left to right: Fc, Fc_1) and MDA-MB-231 (top to bottom: Fc_2, Fc_3, Fc) cells *in vitro.*
(G) MCF7 (top to bottom: 1L, 3L, 3R) and MDA-MB-231 (top to bottom: 13, 32, 22, 1) cells in tumor.

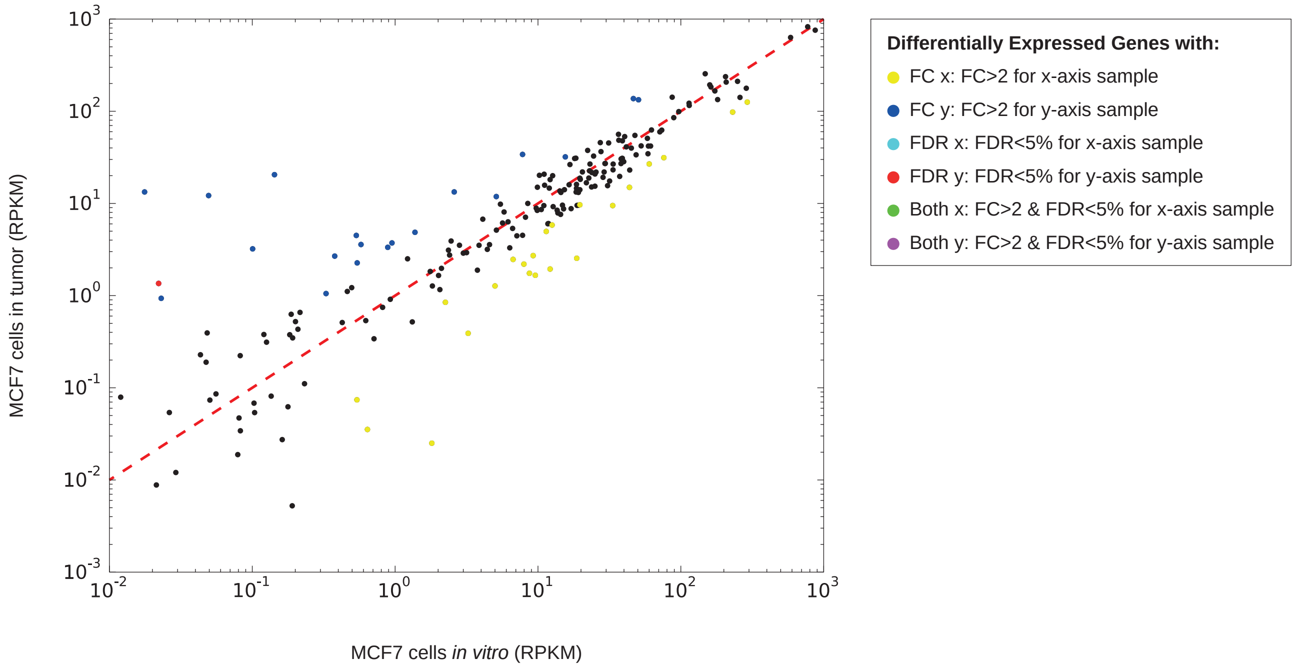

Gene Set:

ADAM17
AKT1
AKT2
AKT3
AP1B1
AP1G1
AP1M1
AP1M2
AP1S1
AP1S2
AP1S3
ARAF
AREG
AREGB
ATF1
ATF2
BCL2
BCL2L1
BMP7
BRAF
BTC
CARM1
CAV1
CAV2
CAV3
CBP
CCND1
CD44
CDH1
CDH3
COL4A1
COL4A2
COL4A3
COL4A4
COL4A5
COL7A1
CREBBP
CSN2
CTNNA1
CTNNA2
CTNNA3
CTNNAL1
CTNNBIP1
CTNND1
CTNND2
DDX5
E2F1
E2F2
EBAG9
EGF
EGFR
ELK1
EMILIN1
EMILIN2
EMILIN3
EP300
EREG
ESR1
ESR2
ESRRA
ESRRB
ESRRG
FGF10
FGFR1
FGFR2
FGFR3
FGFR4
FOS
GJB1
GPER1
GPR182
GRB2
GSK3B
HBEGF
HDAC4
HSP90AA1
HSP90AB1
HSPB1
HSPB2
HSPB3
HSPD1
IGF1
IKBKB
IKBKE
IKBKG
IL6
ITGA2
JUN
KAT3A
KRAS
LAMA5
LDLR
MAPK1
MAPK10
MAPK11
MAPK12
MAPK14
MAPK3
MAPK7
MAPK8
MAPK9
MED1
MMP1
MMP10
MMP11
MMP12
MMP13
MMP14
MMP15
MMP16
MMP17
MMP19
MMP2
MMP20
MMP21
MMP24
MMP25
MMP26
MMP27
MMP28
MMP3
MMP7
MMP8
MMP9
MNAR
MTA3
MYBBP1A
NCOA1
NCOA2
NCOA3
NCOR1
NCOR2
NEDD8
NFKB1
NFKB2
NFKBIA
NFYA
NFYB
NFYC
NOS1
NOS2
NOS3
NR0B2
NRAS
NRIP1
OCLN
PELP1
PIK3C2A
PIK3C2B
PIK3C2G
PIK3C3
PIK3CA
PIK3CB
PIK3CD
PIK3CG
PIK3R1
PIK3R2
PIK3R3
PIK3R4
PIK3R5
PIK3R6
PLCB1
PLCB2
PLCB3
PLCB4
PLCD1
PLCD3
PLCD4
PLCE1
PLCG1
PLCG2
PLCH1
PLCH2
PLCXD1
PLCXD2
PLCXD3
PLCZ1
PRKACA
PRKACB
PRKACG
PRKCA
PRKCB
PRKCD
PRKCE
PRKCG
PRKCH
PRKCI
PRKCQ
PRKCZ
PRL
PRMT1
RAF1
RARA
RARB
RARG
REL
RELA
RELB
RSTS
SAFB
SAFB2
SAP30
SERPINB5
SERPINB6
SERPINB7
SHC1
SHC2
SHC3
SHC4
SMARCA1
SMARCA2
SMARCA4
SMARCA5
SMARCAL1
SMARCB1
SMARCC1
SMARCC2
SMARCD1
SMARCD2
SMARCD3
SMARCE1
SNAI1
SNAI2
SNAI3
SNRK
SOL4A6
SP1
SRC
STAT1
STAT3
STAT5A
STAT5B
STRIP1
STRIP2
STRN
STRN3
STRN4
TGFA
TGFB1
TIMP2
TRAP
TWIST1
TWIST2
UCP1
UCP2
UCP3
VEGFA
VEGFB
VEGFC
ZEB2

Figure S8. Scatterplot of genes in Estrogen-related signaling, differentially expressed in MCF7 cells *in vitro* and MCF7 cells in tumor.

Gene Set Sources:
Marino M, et al. Estrogen Signaling Multiple Pathways to Impact Gene Transcription. Curr Genomics. 2006 Nov; 7(8): 497–508.
Heldring N , et al. Estrogen Receptors: How Do They Signal and What Are Their Targets. Physiological Reviews. 1 July 2007; 87(3): 905-931
Tocris Bioscience. Estrogen Signaling Pathway. 2015. http://www.tocris.com/pathways/estrogenPathway.php#.VTZRkq2qpBc

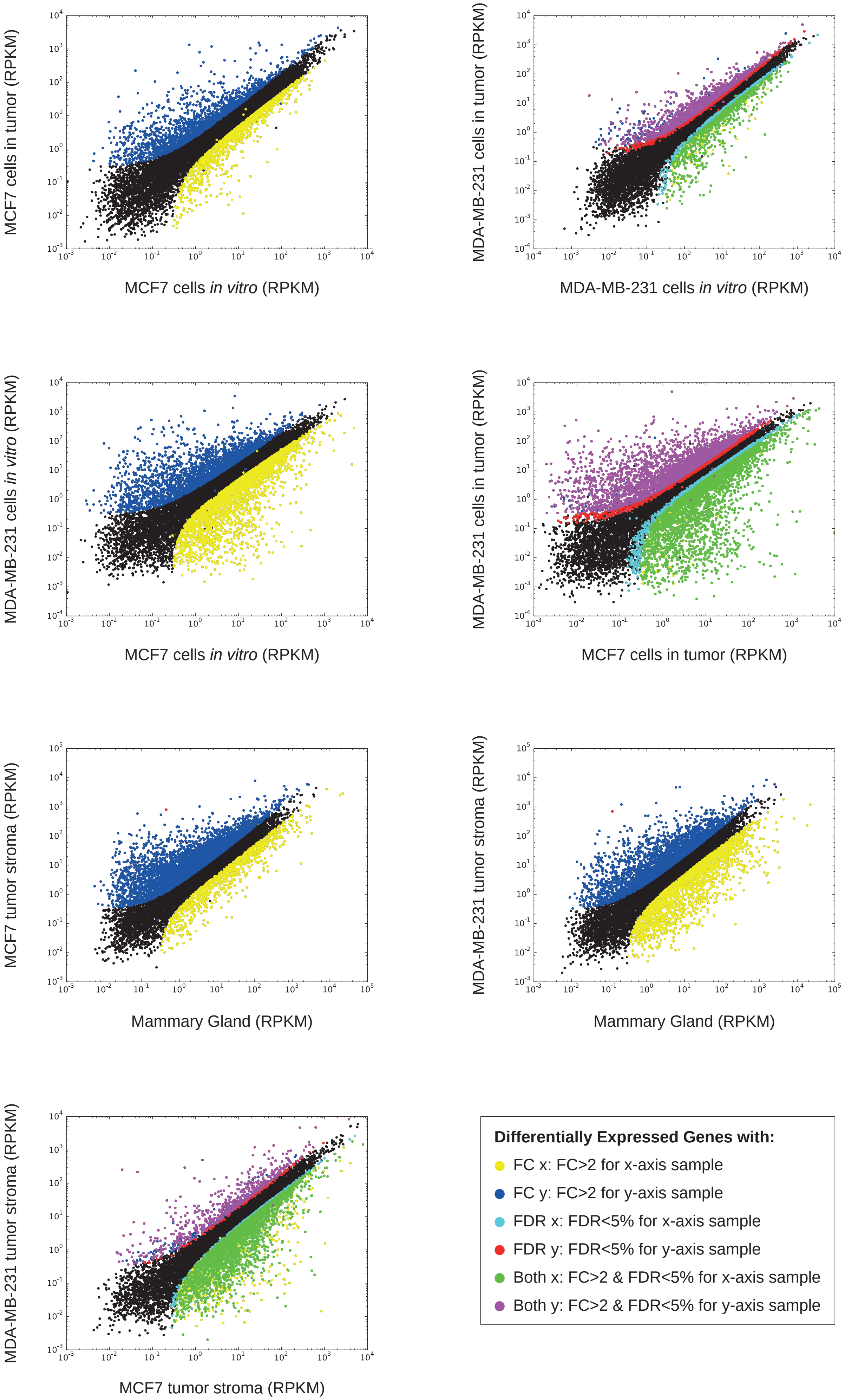

Figure S9. Scatterplots of Table S5 comparison groups.

A. Human expression values:

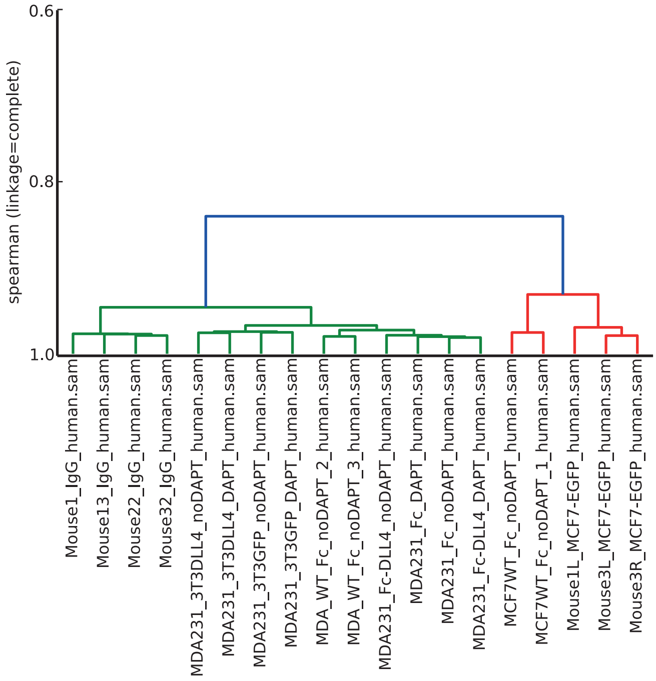

B. Mouse expression values:

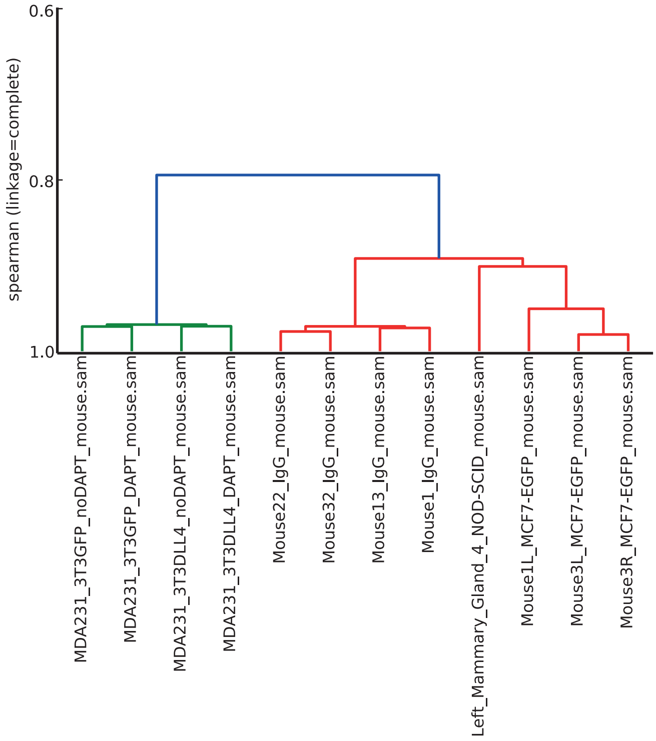

Figure S10. Hierarchical Clustering.
(A) shows human expression values, (B) shows mouse expression values. Human-only samples are only included in (A), mouse-only samples are only included in (B). Clustering is done by complete linkage neighbor-joining of 1-Spearman correlation coefficients.
For human expression values, samples containing MDA-MB-231 cells cluster in green and samples containing MCF7 cells cluster in red. For mouse expression values, *in vitro* samples cluster in green and *in vivo* samples cluster in red. The mammary gland sample clusters more closely with MCF7 tumor stroma than MDA-MB-231 tumor stroma.

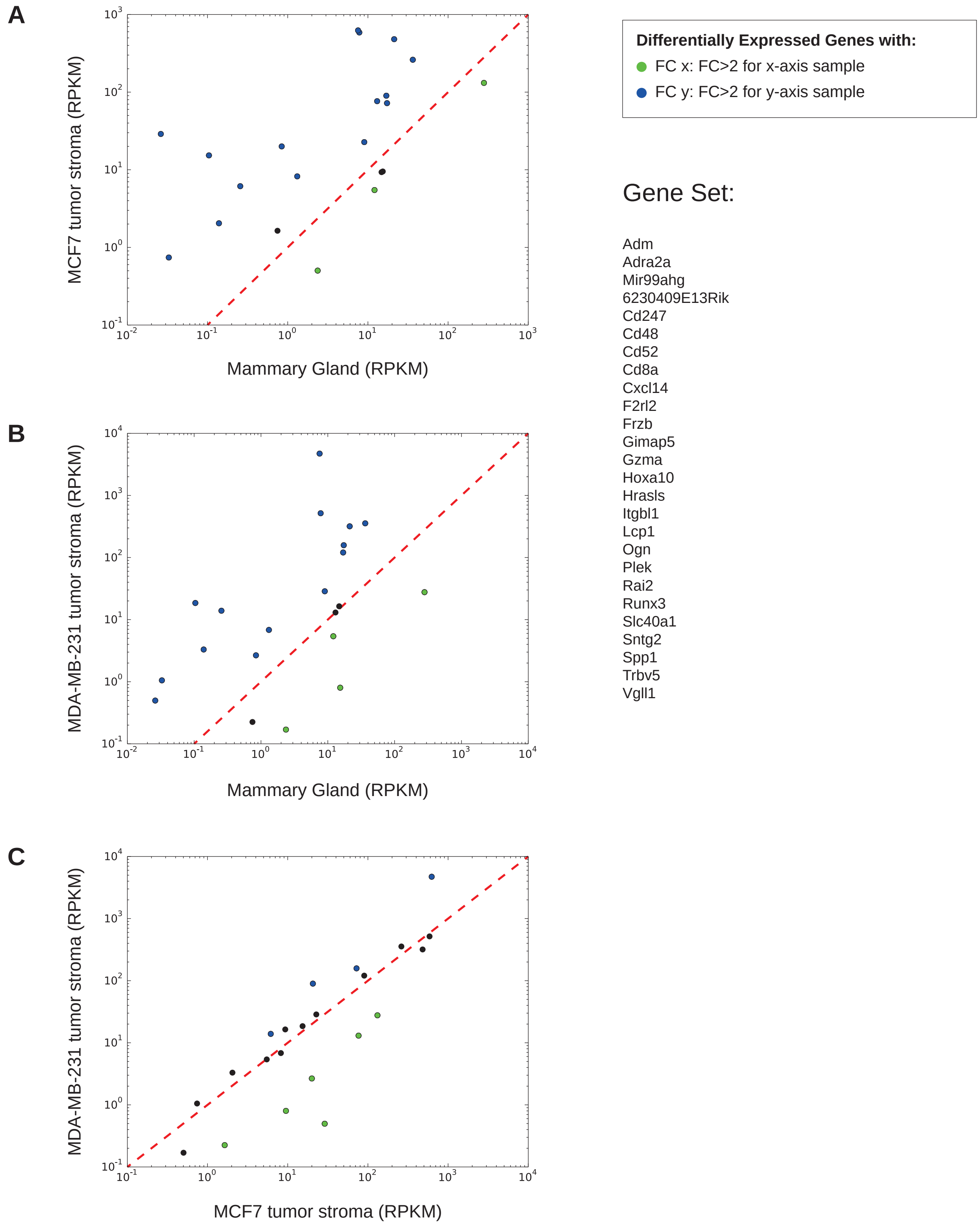

Figure S11. FINAK_BREAST_CANCER_SDPP_SIGNATURE: Scatterplot of genes in the stroma-derived prognostic predictor of breast cancer disease outcome (Finak et al, 2008), differentially expressed in (A) Mammary Gland (MG) compared to MCF7 tumor stroma, (B) MG compared to MDA-MB-231 tumor stroma, and MCF7 tumor stroma compared to MDA-MB-231 tumor stroma.
The Finak_SDPP human gene names were converted to mouse official gene symbols for this study through the use of GeneCards.org (Weizmann Institute of Science), Jax MGI, and NCBI AceView.

Gene Set Sources:
Finak G, et al. Stromal gene expression predicts clinical outcome in breast cancer. Nature Medicine 14, 518 - 527 (2008)
Broad Institute Gene Set Enrichment Analysis, Gene Set: FINAK_BREAST_CANCER_SDPP_SIGNATURE
Mouse Genome Informatics (MGI). The Jackson Laboratory. Mir99ahg. http://www.informatics.jax.org/marker/MGI:1919929
Mouse Genome Informatics (MGI). The Jackson Laboratory. Trbv5. http://www.informatics.jax.org/marker/MGI:98583
AceView. National Center for Biotechnology Information (NCBI). C6orf168. http://www.ncbi.nlm.nih.gov/IEB/Research/Acembly/av.cgi?db=human&c=Gene&l=C6orf168
