# Supplementary material for: Decoding breast cancer tissue–stroma interactions using species-specific sequencing
Source: Breast Cancer Res. 2015 Aug 13;17(1):109. doi: 10.1186/s13058-015-0616-x (PMC4534116; doi:10.1186/s13058-015-0616-x)
Supplement: Additional file 4: Table S3. — Fold change for analysis of Notch activation by co-culture and immobilized ligand. (PDF 410 kb) [file 13058_2015_616_MOESM4_ESM.pdf]

Table S4: Fold change for analysis of Notch activation by co-culture and immobilized ligand.

(A) Comparison of the number of genes upregulated after ligand-activation and downregulated with DAPT treatment, using noted cutoffs, in both co-culture and immobilized ligand assays, (B) gene list of (A) with comparison groups as in Figure 3E and red text indicating repeated gene listing, and (C) comparison of the number of genes up- and downregulated, using noted cutoffs, in MDA-MB-231 cultured with 3T3GFP or Fc.

A

**Comparison: Co-culture vs. Immobilized Ligand**			**Gene Expression**	**FC>2**<br># of Genes
MDA231/3T3DLL4<br>vs. MDA231/3T3GFP (-DAPT)	vs.	MDA231/Fc-DLL4<br>vs. MDA231/Fc (-DAPT)	Upregulated	3
"Notch Signature"<br>in co-culture	vs.	"Notch Signature"<br>on immobilized ligand	In common	1
MDA231/3T3DLL4 (+DAPT)<br>vs. MDA231/3T3DLL4 (-DAPT)	vs.	MDA231/Fc-DLL4 (+DAPT)<br>vs. MDA231/Fc-DLL4 (-DAPT)	Downregulated	10

B

3 common (2 unique) genes upregulated by DLL4 (101 vs. 47 genes)

FAM45B	family with sequence similarity 45, member B (pseudogene)
FAM45B	family with sequence similarity 45, member B (pseudogene)
DYRK4	dual-specificity tyrosine-(Y)-phosphorylation regulated kinase 4

1 common "Notch Signature" gene (63 vs. 29 genes)

SCARNA4	small Cajal body-specific RNA 4

10 common genes downregulated by DAPT (101 vs. 55 genes)

TRIM34	tripartite motif containing 34
SMG1P5	SMG1 pseudogene 5
RNASEK-C17orf49	RNASEK-C17orf49 readthrough
LOC101928837	uncharacterized ncRNA
LOC101928571	ZNF649 antisense RNA 1
INSL4	insulin-like 4 (placenta)
HES4	hes family bHLH transcription factor 4
FRMD8P1	FERM domain containing 8 pseudogene 1
ECSCR	endothelial cell surface expressed chemotaxis and apoptosis regulator
COX7B2	cytochrome c oxidase subunit VIIb2

C

**Comparison: GFP vs. Fc**			**Gene Expression**	**FC>2**<br># of Genes
MDA231/3T3GFP	vs.	MDA231/Fc	Upregulated	352
			Downregulated	97
