# Supplementary material for: Decoding breast cancer tissue–stroma interactions using species-specific sequencing
Source: Breast Cancer Res. 2015 Aug 13;17(1):109. doi: 10.1186/s13058-015-0616-x (PMC4534116; doi:10.1186/s13058-015-0616-x)
Supplement: Additional file 8: Table S7. — Alignment Statistics. (PDF 657 kb) [file 13058_2015_616_MOESM8_ESM.pdf]

Table S3: Alignment Statistics

The number of normalized reads and fraction of human- and mouse-specific reads in each sequenced sample.

'% expressed genes' is '# remaining genes' in Figure S1, '%reads' is '# reads from species1 after separation' divided by the sum of that value and '# reads from species2 after separation', '#reads' is '# reads from species1 after separation', '% read loss' is one minus the ratio of '# reads from species1 after separation' to '# reads from species1 before separation' and '% remaining genes' is '# remaining genes' divided by '# genes before separation'. The values that correspond to the 0.1-0.3% species misassignment frequency are marked in green.

A

in silico

			Sample Information					
			% expressed genes		% reads		# reads	
Sample Description	Species	Sample ID	human	mouse	human	mouse	human	mouse
Human skin 1, SRX800842	human	H1	82,20	6,53	99,86	0,14	20888463	28262
Human skin 2, SRX800868	human	H2	80,30	8,93	99,80	0,20	42072767	84091
Human skin 3, SRX800836	human	H3	79,66	6,03	99,83	0,17	16586093	27870
Mouse skin 1, ERX940616	mouse	M1	13,81	64,81	0,23	99,77	19773	8748660
Mouse skin 2, ERX940615	mouse	M2	17,23	63,10	0,29	99,71	20024	6768565
Mouse skin 3, ERX940617	mouse	M3	13,02	67,13	0,27	99,73	13155	4794321

B

	Alignment Information			
	% read loss		% remaining genes	
Sample ID	human	mouse	human	mouse
H1	16,41	99,48	98,58	10,28
H2	9,15	99,15	98,94	14,29
H3	16,22	99,40	98,75	9,86
M1	98,64	5,24	27,06	99,46
M2	98,36	5,59	34,29	99,23
M3	98,98	5,99	22,22	99,52

C

in vitro

			Sample Information					
			% expressed genes		% reads		# reads	
Sample Description	Species	Sample ID	human	mouse	human	mouse	human	mouse
Notch1 receptor-expressing cells -ligand on cell, -inhibitor	mixed	MDA231_3T3GFP_-DAPT	63,79	63,26	45,78	54,22	5535797	6556909
Notch1 receptor-expressing cells -ligand on cell, +inhibitor	mixed	MDA231_3T3GFP_+DAPT	63,74	63,57	44,37	55,63	5482753	6874231
Notch1 receptor-expressing cells +ligand on cell, -inhibitor	mixed	MDA231_3T3DLL4_-DAPT	63,13	62,57	46,95	53,05	6132342	6928394
Notch1 receptor-expressing cells +ligand on cell, +inhibitor	mixed	MDA231_3T3DLL4_+DAPT	63,61	62,53	47,40	52,60	6294273	6983919
Notch1 receptor-expressing cells -ligand immobilized, -inhibitor	human	MDA231_Fc_-DAPT	66,31	20,43	99,90	0,10	11728605	11692
Notch1 receptor-expressing cells -ligand immobilized, +inhibitor	human	MDA231_Fc_+DAPT	65,73	18,36	99,91	0,09	10818709	9902
Notch1 receptor-expressing cells +ligand immobilized, -inhibitor	human	MDA231_Fc-DLL4_-DAPT	65,73	18,53	99,91	0,09	11265842	10021
Notch1 receptor-expressing cells +ligand immobilized, +inhibitor	human	MDA231_Fc-DLL4_+DAPT	66,03	19,22	99,91	0,09	11661368	10945

D

	Alignment Information			
	% read loss		% remaining genes	
Sample ID	human	mouse	human	mouse
MDA231_3T3GFP_-DAPT	30,98	23,13	92,65	92,89
MDA231_3T3GFP_+DAPT	31,96	22,36	92,61	92,76
MDA231_3T3DLL4_-DAPT	30,01	23,94	92,56	92,61
MDA231_3T3DLL4_+DAPT	29,67	24,15	92,65	92,39
MDA231_Fc_-DAPT	6,77	99,62	99,02	35,64
MDA231_Fc_+DAPT	6,76	99,66	98,92	32,37
MDA231_Fc-DLL4_-DAPT	6,74	99,66	98,94	32,60
MDA231_Fc-DLL4_+DAPT	6,70	99,65	98,93	33,71

E

			Sample Information					
			% expressed genes		% reads		# reads	
Sample Description	Species	Sample ID	human	mouse	human	mouse	human	mouse
MCF7 cells -ligand immobilized, -inhibitor	human	MCF7WT_Fc	63,87	4,91	99,96	0,04	5359310	2234
MCF7 cells -ligand immobilized, -inhibitor	human	MCF7WT_Fc_1	67,10	6,85	99,96	0,04	11092079	4582
MDAMB231 cells -ligand immobilized, -inhibitor	human	MDA_WT_Fc_2	67,69	7,58	99,96	0,04	13826522	5665
MDAMB231 cells -ligand immobilized, -inhibitor	human	MDA_WT_Fc_3	66,30	6,09	99,96	0,04	11091367	3916

F

	Alignment Information			
	% read loss		% remaining genes	
Sample ID	human	mouse	human	mouse
MCF7WT_Fc	11,49	99,85	98,63	9,16
MCF7WT_Fc_1	8,34	99,85	99,07	12,11
MDA_WT_Fc_2	7,67	99,84	99,13	13,31
MDA_WT_Fc_3	7,13	99,87	99,16	10,89

G

in vivo

			Sample Information					
			% expressed genes		% reads		# reads	
Sample Description	Species	Sample ID	human	mouse	human	mouse	human	mouse
MCF7-EGFP in nude mouse, left tumor	mixed	Mouse1L_MCF7-EGFP	66,27	75,53	27,82	72,18	5499836	14266339
MCF7-EGFP in nude mouse, right tumor	mixed	Mouse1R_MCF7-EGFP	53,85	73,95	3,27	96,73	409957	12138342
MCF7-EGFP in nude mouse, left tumor	mixed	Mouse3L_MCF7-EGFP	71,28	69,84	74,79	25,21	15840262	5338921
MCF7-EGFP in nude mouse, right tumor	mixed	Mouse3R_MCF7-EGFP	68,36	73,32	48,27	51,73	9680756	10374075
MDA-MB-231 tumor + IgG	mixed	Mouse1_IgG	71,96	66,12	86,35	13,65	32426279	5125434
MDA-MB-231 tumor + IgG	mixed	Mouse13_IgG	71,26	65,41	89,77	10,23	35026428	3990529
MDA-MB-231 tumor + IgG	mixed	Mouse22_IgG	73,34	69,58	80,45	19,55	33846241	8225212
MDA-MB-231 tumor + IgG	mixed	Mouse32_IgG	70,28	66,91	82,81	17,19	18270756	3792521

H

	Alignment Information			
	% read loss		% remaining genes	
Sample ID	human	mouse	human	mouse
Mouse1L_MCF7-EGFP	43,48	17,76	87,63	97,28
Mouse1R_MCF7-EGFP	88,48	7,56	78,51	98,63
Mouse3L_MCF7-EGFP	15,48	46,78	92,96	93,97
Mouse3R_MCF7-EGFP	27,07	25,16	90,18	96,17
Mouse1_IgG	9,35	61,78	94,53	91,50
Mouse13_IgG	8,22	67,74	94,78	91,24
Mouse22_IgG	10,76	50,88	94,32	93,39
Mouse32_IgG	10,81	55,23	94,04	92,96

I

			Sample Information					
			% expressed genes		% reads		# reads	
Sample Description	Species	Sample ID	human	mouse	human	mouse	human	mouse
4th left mammary gland from NOD-SCID mouse	mouse	Left_Mammary_Gland_4_NOD-SCID	25,19	71,39	0,14	99,86	17809	12925494

J

	Alignment Information			
	% read loss		% remaining genes	
Sample ID	human	mouse	human	mouse
Left_Mammary_Gland_4_NOD-SCID	99,46	5,78	40,96	99,34
