# Supplementary material for: Decoding breast cancer tissue–stroma interactions using species-specific sequencing
Source: Breast Cancer Res. 2015 Aug 13;17(1):109. doi: 10.1186/s13058-015-0616-x (PMC4534116; doi:10.1186/s13058-015-0616-x)
Supplement: Additional file 9: Table S8. — Fold change and false discovery rate for analysis of gene expression in tumor and stroma. (PDF 426 kb) [file 13058_2015_616_MOESM9_ESM.pdf]

A

Comparison			Gene Expression	FC>2 # of Genes	FDR<5% # of Genes	FC>2 & FDR<5% # of Genes
MCF7 cells in tumor (n=3)	vs.	MCF7 cells *in vitro* (n=2)	Upregulated	2142	1	1
			Downregulated	1564	0	0
MDA-MB-231 cells in tumor (n=4)	vs.	MDA-MB-231 cells *in vitro* (n=2)	Upregulated	1456	1770	1112
			Downregulated	897	1189	717
MDA-MB-231 cells *in vitro* (n=2)	vs.	MCF7 cells *in vitro* (n=2)	Upregulated	2935	1	1
			Downregulated	3459	0	0
MDA-MB-231 cells in tumor (n=4)	vs.	MCF7 cells in tumor (n=3)	Upregulated	3562	4635	3388
			Downregulated	3709	4609	3479

B

Comparison			Gene Expression	FC>2 # of Genes	FDR<5% # of Genes	FC>2 & FDR<5% # of Genes
MCF7 tumor stroma (n=3)	vs.	Mammary Gland (n=1)	Upregulated	7123	1	1
			Downregulated	959	0	0
MDA-MB-231 tumor stroma (n=4)	vs.	Mammary Gland (n=1)	Upregulated	4090	1	1
			Downregulated	2171	0	0
MCF7 tumor stroma (n=3)	vs.	MDA-MB-231 tumor stroma (n=4)	Upregulated	4394	3598	3113
			Downregulated	743	736	609

Table S5: Fold change and false discovery rate for analysis of gene expression in tumor and stroma.
A comparison of the number of genes up- or downregulated, using noted cutoffs, in cells (A) and stroma (B).
As false discovery rate (FDR) is influenced by the number of samples in a comparison group, and as the sample group size (n) differs between comparison groups, gene expression with a fold-change (FC) greater than 2 was used for analyses in this study.

For the algorithm which generated these FDR values, both comparison groups cannot have n=1, but one group with n=1 can be compared with another group with n>1. For these latter comparisons, the FDR variations come from the n>1 group rather than the n=1 group.
